# The evaluation of devaluation: Deficient outcome devaluation leads to wrongly considering goal-directed actions as habits

**DOI:** 10.3758/s13428-026-03099-6

**Published:** 2026-07-07

**Authors:** Antonio Vázquez-Millán, Pablo Martínez-López, María Rueda, José J. León, David Luque

**Affiliations:** 1 Department of Basic Psychology, Faculty of Psychology and Speech Therapy, University of Malaga, C/. Doctor Ortiz Ramos, 12, 29010 Málaga, Spain; 2 The Malaga Biomedical Research Institute and Nanomedicine Platform-IBIMA , BIONAND Platform, Málaga, Spain

**Keywords:** Devaluation, Goal-directed, Habit, Learning, Validity

## Abstract

Habits are stimulus-driven responses that are produced independently of the current outcome value. They enable efficient actions in familiar contexts while freeing up cognitive resources. Habits are expected to influence behavior after substantial experience, whereas limited training leaves behavior under goal-directed control. This transition from goal-directed to habitual control has been documented in animal research but remains challenging to replicate in humans. Using a free-operant task, recent studies have suggested that human habits can be fully learned even after short training. This would produce a ceiling effect, explaining null results when the amount of training is manipulated. Here, we propose an alternative explanation: the devaluation protocol was ineffective for a subset of participants who appeared “habitual”. To test this hypothesis, we conducted a preregistered conceptual replication of the free-operant task with improved devaluation. As in previous research, we found no difference in habitual responses between short and extended training. In contrast to prior reports, most participants were sensitive to outcome devaluation. Habitual responding was strongly correlated with the effectiveness of the outcome devaluation protocol; habit-like responses were mostly produced by the few participants for whom the devaluation did not work. We further supported our hypothesis by reanalyzing previous datasets, finding that, across all studies, participants who showed more habit-like responses were those for whom the devaluation was less effective. Taken together, our findings suggest that suboptimal outcome devaluation protocols may have biased previous results, leading to habit-like goal-directed responses regardless of the amount of previous instrumental training.

**Supplementary Information:**

The online version contains supplementary material available at 10.3758/s13428-026-03099-6.

Instrumental human behavior depends on the interaction between two neurocognitive systems: the goal-directed and the habitual systems (Balleine & Dezfoulli, 2019). Goal-directed actions are planned and performed to achieve desired outcomes; when the motivational value of an outcome changes, the behavior typically changes as well. In contrast, when actions are repeated within specific and stable contexts, they can become habits. By relying on habits, organisms reduce the cognitive demands of goal-directed processing and act more efficiently. Once formed, habits lack flexibility and can persist even when their consequences are no longer desired (Wood & Rünger, 2016).

Much of what we know about the functioning of the habit system comes from animal experiments using the outcome devaluation paradigm. Since they rely on stimulus–response (S-R) associations, habits are triggered by a discriminative stimulus or context, and are expressed even if the current outcome value (O) is no longer desired. For example, after extensive instrumental training in an S-R-O chain (e.g., pressing a lever to obtain food), if the response becomes insensitive to reinforcer devaluation (e.g., the food is poisoned), it is considered a habit (Kosaki & Dickinson, 2010). In contrast, if animals adjust their behavior according to the new outcome value, the behavior remains under goal-directed control (Watson et al., 2022).

Thus, learning theories posit that the amount of instrumental training is a crucial factor in maintaining a balance between goal-directed and habit systems. While this prediction has collected some favorable evidence in nonhuman animal research (Killcross & Coutureau, 2003; Adams, 1982), translating those laboratory procedures to humans has proven challenging (Watson & de Wit, 2018). Although habitual processing has been widely investigated in humans (for a review, see Guida et al., 2022), researchers have struggled to demonstrate the expected transition from goal-directed to habitual control based on the amount of training. Indeed, a series of experiments have failed to show an increase in outcome-insensitive responses after overtraining compared to less-trained conditions (de Wit et al., 2018). It has been suggested that these results may be explained by the fact that human executive control can inhibit even strongly ingrained habits in favor of goal-directed behavior (Buabang et al., 2025; Littman et al., 2019). Based on this idea, recent experimental protocols include elements to deactivate or hinder executive control. For instance, the *forced-response task* revealed habitual responding when participants were forced to respond rapidly; notably, the same individuals showed goal-directed control when they had longer response preparation times (Hardwick et al., 2019; Martínez-López et al., 2026; see also Luque et al., 2020). Critically, this evidence suggests that it would be unexpected to find habitual actions in humans using standard experimental protocols in which participants have no constraints on the operation of their executive system during habit tests.

The study by Tricomi et al. (2009) is a rare case in which increased habitual responding was found after overtraining using a free-operant task inspired by animal paradigms (of note, this experiment was conducted while participants were scanned in a magnetic resonance imaging [MRI] system; we do not discuss the neuroimaging results here; see Gera et al., 2023, for details). Participants learned to associate fractal images with key presses to earn two different food rewards, which were provided at the end of the experiment. Crucially, responses were self-paced throughout the trial thereby without time pressure or other goal-directed constraints. Devaluation was achieved through selective satiation, in which participants consumed one of the foods until it became unappealing, thereby supposedly reducing or suppressing their motivation to obtain the devalued outcome. After devaluation, the short-trained group (1 day of training) reduced their response rate during the habit test, whereas the overtrained group (3 days of training) continued to press the key associated with food, despite being supposedly sated.

Tricomi et al. (2009) was the first study successfully capturing changes in habit expression due to overtraining, as predicted by learning theory. In addition, their task was very similar in key aspects (free-operant schedule, food as outcomes, devaluation via satiation) to animal paradigms. This is important because it suggests that animal protocols could serve as a valid tool for studying the habit system in humans, facilitating translational endeavors. However, three studies have since failed to replicate these crucial findings (de Wit et al., 2018; Gera et al., 2023; Pool et al., 2022). Firstly, de Wit et al. (2018) conducted two experiments using a very similar task but found that participants still maintained outcome-sensitive behavior—indicative of goal-directed control—regardless of the amount of instrumental training.

Pool et al. (2022) conducted a multisite study to investigate the discrepant results between the original Tricomi et al. (2009) study and those of de Wit et al. (2018). Their preregistered analyses showed similar reductions in responses to devalued outcomes across both training conditions after the satiation procedure, replicating the null results reported by Wit et al. (2018). Unlike previous studies, Gera et al. (2023) incorporated MRI scanning to attempt a direct replication of Tricomi’s original protocol to investigate habit formation. This addition could have been critical, as MRI procedures can induce stress, potentially impairing the cognitive resources needed for goal-directed actions and promoting habit learning (Schwabe & Wolf, 2011). Nevertheless, the authors reported results almost identical to those of Pool et al. (2022): reductions in response rates to the devalued outcome were similar across both training conditions. We think the most straightforward explanation for this pattern of results is that the paradigm lacked cognitive constraints, favoring goal-directed control. Therefore, those participants who found the food truly aversive after the devaluation were capable of inhibiting habitual responding, independently of how much that response had been trained.

Nonetheless, Pool et al. (2022) suggested an alternative explanation primarily based on a set of non-preregistered cluster analyses. These authors found that most participants in both training groups did not adjust their responses after devaluation, suggesting behavior consistent with habitual control. Pool et al. (2022) suggested that this experimental paradigm was so effective at inducing habits that even a single day of training was sufficient for many participants to form new habits. As a result, manipulating training duration had little effect, since behavior was already largely habitual after short training, leaving little room for further habitualization with extended training. Their exploratory analyses regarding individual differences also revealed that those participants reporting higher levels of affective stress were more likely to display outcome insensitivity (i.e., habit-like behavior). The authors conclude that this protocol was a valid tool for assessing habits and that it offers a promising approach for studying individual differences in habit formation processes.

Following the same approach, Gera et al. (2023) performed cluster analyses that similarly showed the overall result was driven by a small subset of participants, as most in both groups did not adjust their responses after devaluation. Again, the authors concluded that the task effectively induces habits even after short training. While it may not capture the effects of training duration on habitual control, it would remain a valuable tool for studying the habit system without requiring extended practice.

Therefore, we stand before two irreconcilable positions to explain the absence of differences in habitual responses between different training conditions: we believe it reflects that the task is not valid for measuring habits, and habit-like responses are due to inadequacies in the devaluation method, whereas other authors suggest the null result is a consequence of the experimental paradigm being extremely powerful to create habits even with minimal training periods.

From our perspective, there are reasons to remain skeptical about the conclusions drawn by Pool et al. (2022) and Gera et al. (2023). From a theoretical perspective, these claims are not well aligned with the computational principles of habit formation (Marr, 1982). The computational model posits that habits complement goal-directed decision-making because they enable organisms to act efficiently (e.g., rapidly and with minimal cognitive effort), while they are mainly in stable, low-risk contexts. If habits were learned too quickly, they could form in unstable situations, leading to maladaptive, perseverative behaviors. In other words, the habit system would lose its adaptive function.

Moreover, the predominant view holds that habits are behavioral tendencies rooted in stimulus–response (S-R) associations (Verplanken & Orbell, 2022). These S-R memories are created and strengthened through repeated experiences where actions are rewarded within their associated stimulus context (Wood et al., 2022). Thus, the amount of training should be a reliable predictor of habitual control. Even if Tricomi’s task could induce habits after a single training session, the resulting memory trace should be weaker than that formed after 3 days of training, and a robust habit test should detect such differences. The alternative—habits formed after brief training already reach maximum strength—seems unlikely, as it would imply that we form strong, rigid habits with minimal experience, a notion fundamentally at odds with the core concept of habits.

Therefore, we propose a less radical alternative explanation for the results from the studies by Pool et al. (2022) and Gera et al. (2023). Our hypothesis aligns with the aforementioned idea that participants are under goal-directed control throughout the task (regardless of the amount of training). We think that shortcomings in the outcome devaluation procedure (i.e., selective satiation procedure) led to confounding goal-directed actions with habits. If the devaluation was not fully effective, some participants could intentionally pursue the outcome (i.e., the food), which was wrongly considered as devalued. Thus, for some participants, behavior was misinterpreted as habitual when, in fact, it was still operating under goal-directed control. Our hypothesis is that these participants would be the majority in a seemingly “habitual” cluster when a cluster analysis is conducted on such data.

There are some factors in the outcome devaluation protocol used by Tricomi et al. (2009) and its replication attempts that we think could hinder its efficacy. Despite the researchers’ instructions and even after being sated, participants know they will not be forced to eat the rewards they earn during the habit test. This creates a dissociation between the earned outcome and the actual aversive experience of eating when full (i.e., obtaining the outcome may not be disgusting at all). It raises doubts about whether responses linked to the sated food are genuinely perceived as undesirable, that is, whether the motivational value of the outcome has genuinely been devalued (de Houwer et al., 2018). In fact, participants may continue responding in a goal-directed manner because they want to earn the food to eat later or believe that accumulating more rewards will improve their performance in the experiment (Eder & Dignath, 2016, 2019). In this regard, some participants might refrain from eating until they are completely sated due to fear of being judged or simply wanting to finish the experiment quickly. Others might keep pressing keys out of boredom, as an alternative to doing nothing. Clearly, none of these scenarios reflects genuine habit expression.

Notably, these studies included manipulation checks to verify the effectiveness of the devaluation. Participants rated their hunger and the pleasantness of each food on a Likert scale before and after consuming the food intended for devaluation. As reported, the overall pleasantness ratings for the devalued outcome (i.e., the food eaten to satiation) significantly decreased following the procedure (Gera et al., 2023; Pool et al., 2022), supporting the expected effect of satiation. While manipulation checks are widely used to ensure the validity of experimental manipulations (Shadish et al., 2002), they can be poorly informative in some cases (Ejelöv & Luke, 2020). For instance, relying solely on group-level significant differences does not inform us about the extent to which a certain manipulation was effective at the individual level. This leaves room for concerns about its validity, especially when researchers want to make claims at the individual level (e.g., some individuals are under habitual control, others are not). Thus, even if we accept the satiation protocol as an effective method for devaluing the outcome, its effect should be assessed at the individual level, not just the group level (Molinero et al., 2025).

A significant group-level decrease in pleasantness ratings does not guarantee that devaluation was successful for every participant; the effect could be driven by a small subset of participants. Participants who were not effectively sated would be erroneously classified as under habitual control when, in fact, their responses remained goal-directed (i.e., they still perceived the food as pleasant and desirable). In this vein, Gera et al. (2023) reported in their supplemental materials that the effectiveness of the satiation protocol, as measured by pleasantness ratings, was correlated with the behavioral index of habits. Participants for whom the devaluation was less effective also showed more habit-like responses. This finding supports the idea that participants within the outcome-insensitive cluster were actively pursuing the food reward because the devaluation protocol failed to entirely reduce its motivational value.

Lastly, an optimal devaluation procedure should selectively affect the target outcome, while leaving the motivational value of the other outcome(s) relatively intact to allow meaningful comparisons. However, satiation procedures have been criticized for lacking this specificity, as they can partially reduce the value of both outcomes (Smeets et al., 2023). This undermines subsequent analyses comparing response rates to cues linked with the satiation versus non-satiation food outcomes.

In sum, the claim that habits emerged after short training for a subset of participants is called into question by issues with the devaluation protocol and manipulation checks. The overarching objective of the current study is to further investigate how the efficacy of the devaluation procedure affects behavioral performance on the habit test. We confront our hypothesis, focused on devaluation shortcomings, with the alternative explanation of habit formation after just 1 day of training in a free-operant task, as previously suggested by Pool et al. (2022) and Gera et al. (2023). For this, we first conducted a conceptual replication of Pool et al. (2022) and Gera et al. (2023), improving the devaluation protocol. We expect to replicate the null effect of overtraining, but now with a small number of participants who are classified as “habitual” in the cluster analysis (see our preregistered hypothesis). Second, we expect to find that seemingly habitual responses are more frequent among participants whose devaluation was less effective (even with our improved devaluation protocol, there was some variability in its efficacy). This hypothesis was assessed through a set of analyses on our data and data from Pool et al. (2022) and Gera et al. (2023).

Regarding our conceptual replication, the main difference with previous replications of Tricomi et al. (2009) is that we replaced food rewards with monetary rewards. In our protocol, one outcome—initially valued—is devalued to imply a monetary loss, while the other retains its positive value. Notably, this approach should ensure that the devalued outcome consistently acquires an aversive component across participants, overcoming some of the aforementioned drawbacks from previous studies. Unlike satiation, it minimizes the risk that participants continue responding due to alternative goals (e.g., stockpiling food for later) and should prevent individual differences related to subjective factors such as hunger perception or fear of social judgment. Moreover, our devaluation procedure targets only the specified outcome, preserving the motivational value of the alternative, which continues to deliver monetary rewards.

To be clear, we think that finding evidence of habits in humans requires more refined methods that somehow hinder the efficiency of human goal-directed processes (see Watson et al., 2022; Wood et al., 2022). Thus, we remain skeptical about the actual validity of the free-operant paradigm for measuring habits in humans. For this reason, in our preregistration, we hypothesized that the amount of training would not affect the behavioral expression of habitual control (Hypothesis 1).

We also preregistered a cluster analysis, following the approach of Pool et al. (2022) and Gera et al. (2023), anticipating that we would find only one cluster of goal-directed participants (Hypothesis 2). Since we attributed the two-cluster solution in previous studies to shortcomings in the selective satiation procedure, we expected that enhancing the effectiveness of outcome devaluation (by employing our money-based protocol) would decrease the occurrence of habit-like behaviors and, consequently, the proportion of outcome-insensitive participants.

In brief, we found no effect of the amount of training on habit expression, confirming Hypothesis 1. The hypothesis regarding cluster analysis, however, warrants reformulation. Contrary to our initial prediction, the best-fitting solution consisted of two clusters; however, the distribution of participants across these clusters did not align neatly with a “goal-directed” versus “habitual” classification: most participants exhibited response patterns consistent with goal-directed control. Thus, despite the result not being as expected, it still supports our hypothesis: previous findings of outcome-insensitive responses could be an artifact arising from caveats of the partial satiation procedure. Therefore, (1) the occurrence of these habit-like behaviors decreased drastically when a more precise devaluation method was used, and (2) at an individual level, habit-like behavior was significantly correlated with the effectiveness of the outcome devaluation protocol.

As mentioned, we further explored our hypothesis by conducting additional non-preregistered analyses that revealed that habit-like behavior was more likely when participants misunderstood the devaluation instructions. Furthermore, in a non-preregistered reanalysis of the original data from Pool et al. (2022) and Gera et al. (2023), we found that habit-like behavior in those datasets was also dependent on the effectiveness of outcome devaluation.

Pool et al. (2022) and Gera et al. (2023) also claimed that participants with higher levels of “stress affect” were more prone to exhibit habitual control of behavior. We were unable to replicate this finding. Overall, our results provide substantial grounds for believing that the perseverative behaviors observed in our study, as well as in Pool et al. (2022) and Gera et al. (2023), were not genuine habits and were unrelated to “stress affect” or other personality traits, at least among those we measured.

## Method

### Transparency and openness

The study adheres to the APA Style Journal Article Reporting Standards (Appelbaum et al., 2018). We preregistered this research on the Open Science Framework (OSF), specifying the experimental paradigm, central hypothesis, and analysis plan (see Preregistered protocol).

We complied with the sample size and data exclusion criteria established in the preregistration. All raw data by participant and the preprocessed databases are available in the public open-access online repository: https://osf.io/tfhea/ (Vázquez-Millán et al., 2025). We also submitted the scripts used to program the computerized task, preprocess data, and conduct statistical analyses. Some materials are adapted versions of previously published open-access works. We explicitly recognized and specified the authorship of these source codes in the following sections.

### Participants

A total of 123 undergraduate students from the University of Málaga completed the experiment. Sixteen participants were excluded because they failed the stimulus–outcome contingency test administered after instrumental training (see Procedure section), in accordance with our preregistered criteria. Specifically, we excluded those who scored zero or below (i.e., in the wrong direction) on the Likert scale measuring how well they linked each fractal with its outcome, as this indicated the associations were likely not learned.

A total of 107 participants were included in the primary analyses. We calculated the sensitivity of our analyses using G*Power software (version 3.1.9.7), focusing on the interaction effect between cue (devalued vs. non-devalued) and group (moderate training vs. overtraining) on instrumental response in a repeated-measures analysis of variance (ANOVA). Cue was treated as a within-subject factor, and group as a between-subject factor. Assuming α = 0.05 and statistical power = 0.90, and incorporating the correlation between the two cue levels (*r*^2^ = −0.17), our 2 × 2 ANOVA was powered to detect a minimum effect size of Cohen’s* f* = 0.242 (*η*_*p*_^2^ = 0.055) for the interaction effect. Notably, two participants did not complete the questionnaires, so analyses involving individual differences were conducted with *N* = 105.

Participants were randomly assigned to one of two training groups: minimal training (*n* = 55) or overtraining (*n* = 52). Table 1 shows the demographic information. There were no significant differences between groups in gender distribution [*χ*^2^(1) = 0.474, *p* = 0.491] or mean age [*t*(72.83) = − 0.933, *p* = 0.354]. We also found no between-group differences in any questionnaire scores (see Supplemental Material, A1). Participants received course credits for their participation and a financial reward ranging from €5 to €15, depending on task performance.

**Table 1:** Descriptive statistics for the sample: gender distribution and mean age by group

	Age *M* (*SD*)	Males *N* (%)	Females *N* (%)	NB *N* (%)	Total
Minimal training (1 day)	19.11 (2.17)	8 (14.55%)	47 (85.45%)	0 (0%)	55
Overtraining (3 days)	19.75 (4.48)	11 (21.16%)	40 (76.92%)	1 (1.92%)	52
Full sample	19.42 (3.49)	19 (17.76%)	87 (81.31%)	1 (0.93%)	107

*NB* nonbinary

All participants provided informed consent prior to commencing the computerized task. Voluntary participation and data anonymity were guaranteed. The procedures and data handling plan were approved by the University of Málaga Ethics Committee (code 46–2020-H).

### Apparatus and questionnaires

Participants were seated in semi-closed cubicles equipped with standard PCs and 38.4 cm monitors, at a viewing distance of approximately 85 cm. Screens had a 60 Hz refresh rate. Stimuli were presented and controlled using MATLAB 2022b (MathWorks, Natick, MA, USA) with Psychophysics Toolbox version 3.0.19.4 (PTB-3; Brainard, 1997; Pelli, 1997; Kleiner et al., 2007). The task code was adapted from the publicly available online version used by Pool et al. (2022) and is accessible in the OSF repository (see https://osf.io/tfhea/).

We administered the Spanish versions of the same questionnaires used by Pool et al. (2022) to replicate their analyses of the moderating effects of individual differences^1^.

#### Trier Inventory for Chronic Stress (TICS)

The TICS (Petrowski et al., 2012) measures the frequency with which individuals have faced complex or stressful situations over the past 3 months, using a 5-point Likert scale (0 = *never*, 4 = *very often*). It includes 57 items grouped into nine dimensions of chronic stress: work overload, social overload, pressure to perform, work discontent, excessive demands at work, lack of social recognition, social tensions, social isolation, and chronic worrying. It also provides an overall screening index of chronic stress. Internal consistency for each dimension was assessed via Cronbach’s alpha and ranged from 0.71 to 0.86 (see Supplemental Material, A2 for details).

#### Barrat Impulsiveness Scale (BIS-11)

The BIS-11 (Patton et al., 1995; Oquendo et al., 2001) assesses impulsiveness as a personality or behavioral trait, examining various patterns of acting and thinking. Items are rated on a 4-point Likert scale (1 = *never or rarely*; 4 = *always or almost always*), with a total score ranging from 30 to 120. The BIS-11 also distinguishes three subcomponents of impulsivity: attentional or cognitive impulsiveness, motor impulsiveness, and non-planning impulsiveness. Internal consistency for the total score was Cronbach’s α = 0.78.

#### State–Trait Anxiety Inventory (STAI)

We employed the STAI-Trait subscale, as we were interested in assessing individuals’ general tendency to experience anxiety in daily life (Spielberger et al., 1971; Guillén-Riquelme & Buela-Casal, 2011). The scale includes 20 items, rated on a 4-point Likert scale (0 = a*lmost never*; 3 = a*lmost always*). Higher scores indicate a greater propensity for anxiety. Internal consistency was Cronbach’s α = 0.88.

#### Obsessive–Compulsive Inventory-Revised (OCI-R)

The OCI-R (Foa et al., 2002; Malpica et al., 2009) measures the typical symptoms of obsessive***–***compulsive disorder and how disturbing they are. Items are rated on a 5-point Likert scale, ranging from 0 (i.e., *not at all*) to 4 (i.e., *very much*). We only used the global score, which ranges from 0 to 24, with higher scores indicating more severe symptoms. Internal consistency was Cronbach’s α = 0.87.

#### Beck Depression Inventory II (BDI-II)

The BDI-II (Beck et al., 1996; Sanz et al., 2003) consists of 21 items describing feelings, thoughts, and behaviors commonly associated with depression. It provides an index of both the presence and severity of depressive symptoms. Participants rate the frequency or intensity of each symptom on a 4-point Likert scale (0 to 3). In the present sample, internal consistency reached a Cronbach’s α = 0.92.

## Experimental procedure

Upon arrival at the laboratory, participants provided written informed consent. They were randomly assigned to one of the training conditions using a simple computerized algorithm based on subject number. The free-operant task then began. This task was adapted from the original open code provided by Pool et al. (2022), and our adapted code is available in the OSF online repository (https://osf.io/tfhea/). The task consisted of three main stages: (1) training, (2) outcome devaluation, and (3) extinction test (also called the *habit test*).

### Training phase

Each training trial presented a fractal image (the discriminative stimulus) alongside four gray mini-squares on the screen. One fractal signaled a rest period, during which participants were instructed not to respond. The two other fractals signaled active trials, where participants could earn outcomes by pressing the correct key. There were four response options (keys “D,” “F,” “J,” and “K”). The correct key for a given fractal was indicated by one mini-square changing color from gray to yellow during that block. Mini-squares were spatially mapped to the keys (e.g., if the leftmost mini-square turned yellow, the correct response was the leftmost key).

**Fig. 1:**
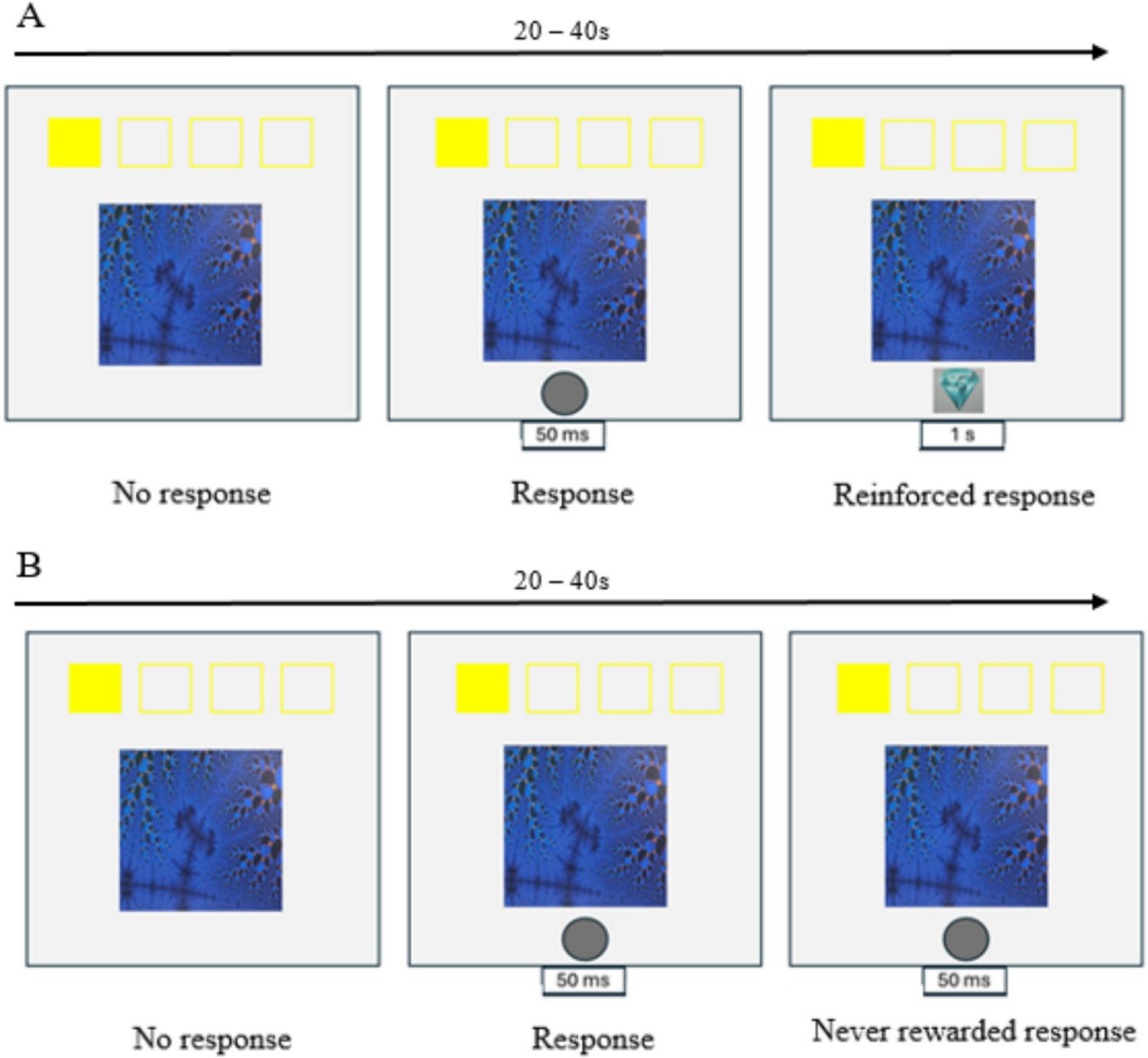
Design of the free-operant task trials during **A** the training phase and **B** the extinction test. *Note*. **A** Active trial during the instrumental learning phase following a 10-s variable interval schedule of reinforcement: there was a 1/10 chance each second of the prize being available. Responses were self-paced, and each trial lasted 20 or 40 s. **B **Active trials during the test phase were conducted in extinction. Responses were also self-paced and recorded, but no rewards were provided. In both phases, active trials were interspersed with baseline trials, during which no colored squares were displayed, responses were not recorded, and outcomes were not available

Responses were self-paced, and rewards were delivered following a variable interval schedule (VI 10 s), meaning there was a 1-in-10 chance each second that a reward would become available. When a response was rewarded, an image of either a diamond or a gold bar was displayed for 1 s, depending on the fractal. If no reward was available, a gray circle briefly appeared (50 ms) each time the correct key was pressed (see Fig. 1). Crucially, each fractal was consistently paired with a specific key response and a specific outcome (diamond or gold bar), and participants were instructed to memorize these associations. The training phase instructions also explained that the total amount of money received at the end of the experiment would increase each time one of these outcome images appeared, with the potential to earn up to €15. Participants were explicitly informed that both outcomes had identical value (i.e., they contributed equally to the final monetary reward). At the end of each block, a message displayed the total number of outcomes earned.

Each training block included 12 active trials, lasting 20 or 40 s (i.e., the duration the fractal remained on screen with rewards available), and eight resting trials of 20 s. Trial order was pseudo-randomized to prevent the same fractal from appearing consecutively. For each participant, fractals were randomly assigned to rewards or to the resting state, and the stimulus–response–outcome (S-R-O) associations remained fixed throughout the experiment.

Before the real training, participants completed a practice block where they learned to press the correct key depending on the colored mini-square; during this practice, the main stimuli and rewards were not presented. Once this practice was finished, the real training started.

The minimal training group (1 day) completed two training blocks on a single day, while the overtraining group (3 days) completed 12 blocks, with four blocks per day across three consecutive days.

### Outcome devaluation

Once the training was complete, participants had to pass the contingency test by rating the probability of obtaining one of two outcomes for each fractal, using an 11-point bipolar scale. The endpoints of the scale represented the two rewards; participants were instructed to move the marker toward the outcome they believed was associated with the fractal shown. If they believed both outcomes were equally probable, they were to leave the marker centered.

Next, participants were informed that one of the rewards was now “cursed,” meaning that from that point on, obtaining that outcome would result in a monetary loss. The following text was displayed: “*THE CRISIS IS HERE! From now on, acquiring GOLD will REDUCE your MONEY”* (Spanish original text: *“¡LLEGÓ LA CRISIS! Desde este momento conseguir ORO te RESTARÁ DINERO”*). This step constituted the outcome devaluation procedure. To ensure participants understood the change, they were asked to rate how pleasant or unpleasant they found each outcome and fractal using an 11-point Likert scale (−5 = *very unpleasant*; 5 = *very pleasant*).

### Extinction test

After completing the pleasantness evaluation, participants were informed that the task was about to restart. Apart from the previous warning about the “curse” affecting one of the outcomes, no further information was provided. The habit test was conducted under extinction conditions to prevent new learning and took place immediately after outcome devaluation. The trial structure was identical to the training phase, except that no rewards were delivered (although the gray circle still appeared with each response). The test included six active trials (three per fractal associated with an outcome) and three resting trials, presented in a pseudo-randomized order to ensure the same fractal did not appear in consecutive trials.

Participants in the minimal training group completed these phases on their first and unique experimental day, while the overtraining group completed them during the third and final session (the first two sessions consisted solely of training blocks).

The response rates toward the devalued and still-valued outcomes were compared after correcting for baseline response rates measured during training. If the response rate toward the devalued outcome remains similar to the baseline, as it should for the still-valued outcome, the behavior is considered to be under habitual control.

Given the length of the experimental sessions (approximately 30 min), the questionnaires were administered online to minimize fatigue-related effects. After completing the free-operant task, participants received an email with a survey link. The survey remained available for 2 weeks, but participants were encouraged to complete it as soon as possible, with reminders sent every few days. Importantly, participants were informed that completing the questionnaires was required to receive the monetary reward they had earned during the free-operant task.

## Statistical analyses

Statistical analyses were conducted using the R programming language (RStudio version 4.4.0). We used the openly available code from Pool et al. (2022) to replicate their analyses and facilitate comparison of results. As part of our preregistered analyses, we performed repeated-measures ANOVAs, combining a between-subjects factor [training (minimal vs. overtraining)] and a within-subject factor [cue (devalued vs. non-devalued)]. Although the dependent variables did not follow a normal distribution, this was not considered a major concern, as previous simulation studies have shown that this type of ANOVA is robust to non-normality provided the sphericity assumption is met (Blanca et al., 2023). When the sphericity assumption was violated, degrees of freedom were adjusted using the Greenhouse–Geisser correction. At other stages, we introduced several modifications to adapt the code to our specific needs and to reanalyze the data from Pool et al. (2022) and Gera et al. (2023).

The significance level was set at *p* < 0.05. For preregistered analyses, we used Tukey’s honestly significant difference (HSD) method when necessary to control for Type I error in multiple post hoc comparisons. For exploratory analyses, we initially conducted tests without adjustment; if any significant results emerged, we repeated the analyses applying the HSD correction. Our analysis scripts are available in the OSF online repository (see https://osf.io/tfhea/).

### Preregistered analyses

#### **Manipulation check**

We compared the liking ratings for each outcome and fractal at the group level to replicate the manipulation check procedures used in previous studies. A 2 × 2 repeated-measures ANOVA [cue (devalued vs. non-devalued) × training (minimal vs. overtraining)] was conducted to confirm that the devalued outcome and its associated fractal were rated as significantly less pleasant, to a similar degree, across both training groups.

#### **Outcome devaluation changes**

Following the original analysis, we calculated a differential index by subtracting the post-devaluation response rate (from the extinction test, which consisted of three trials per outcome) from the pre-devaluation response rate (from the last six trials per outcome during training). This differential score served as the dependent variable in a 2 × 2 repeated-measures ANOVA (cue [devalued vs. non-devalued] × training [minimal vs. overtraining]). We were especially interested in the interaction effect to test whether sensitivity to changes in outcome value varied across training durations. We reported partial eta-squared (*η*^2^*ₚ*) as the effect size for ANOVA contrasts and Cohen’s* d* for post hoc mean comparisons. Additionally, we calculated Bayes factors (*BF*_10_) to quantify the evidence for the alternative hypothesis (i.e., that press frequency differences between cues depend on training group) relative to the null, using Bayesian ANOVAs (Rouder et al., 2012).

#### **Cluster analyses**

Cluster analyses were conducted using the *FlexMix* R package (Leisch, 2004), following the approach of Pool et al. (2022). We first calculated the behavioral adaptation index (BAI) by subtracting the behavioral pre–post change of the valued outcome from the behavioral pre–post change of the devalued outcome [(valued cue post-devaluation − valued cue pre-devaluation) − (devalued cue post-devaluation − devalued cue pre-devaluation)]. Positive BAI values indicated goal-directed behavior, reflecting greater reductions in responses to the devalued cue compared with the valued one. A BAI near zero suggested outcome-insensitive (habit-like) behavior, with similar response rates for both outcomes regardless of the devaluation. Negative BAI values were considered unexpected, indicating a larger decrease for the valued cue than the devalued one. We estimated models ranging from one to five latent clusters, repeating each iteration 500 times to ensure solution stability. We then compared models and selected the best-fitting solution for the data distribution of the behavioral adaptation index, based on the Bayesian information criterion (BIC).

#### **Moderating effects of individual differences**

We first extracted the subscale scores from the questionnaires and submitted them to an exploratory factor analysis (EFA) using the *psych* R package (Revelle, 2017). Analyses were conducted using maximum likelihood estimation with *oblimin* rotation, aiming to identify the factorial solution that best fit our self-report measures. Subscale loadings onto each factor were obtained through the regression method. To assess robustness, we also repeated the EFA using *varimax* rotation, as reported by Pool et al. (2022) in their supplemental material.

After determining the EFA solution, we included the resulting composite factor scores in a linear mixed model (LMM) analysis, alongside the fixed effects of phase (pre- vs. post-devaluation), cue (valued vs. devalued), and training condition (minimal vs. overtraining). Our goal was to test whether these variables interacted to influence response rates during the free-operant task. The trial order was also included as a fixed effect. For all models fitted in this paper, categorical predictors were coded using deviation coding. Following Barr (2013), we built the random effects structure for participants and selected the model with the maximal feasible random effects structure that did not lead to convergence issues (Matuschek et al., 2017). In this case, intercepts for participants and by-participant random slopes for the main and interaction effects of cue and phase were included as random effects to control interindividual variability. Analyses were conducted using the *lmer4* R package (Bates et al., 2015).

It is worth noting that we excluded the OCI-R and BDI from these preregistered analyses to align with the approach of Pool et al. (2022). Although these measures were part of the original plan, the authors ultimately excluded them because only a few study sites had administered the questionnaires. In a second step, we repeated the complete set of analyses, this time adding the OCI-R and BDI scores to examine whether these symptoms influenced the expression of habitual behavior. As this additional analysis did not reveal meaningful differences from the original results, we have reported it in the Supplemental Material (B3).

### Exploratory analyses

#### **Relationship between devaluation efficacy and changes in response rate following devaluation**

We hypothesized that changes in response rate after devaluation were conditioned on participants’ correct understanding of the instructions regarding the loss of value for one of the outcomes. Differences in pleasantness ratings between the two outcomes after the devaluation warning served as an indicator of this understanding—and, by extension, of devaluation efficacy. Specifically, participants who correctly understood the instructions should rate the devalued outcome as unpleasant while maintaining a positive evaluation of the still-valued outcome. Any other pattern would suggest a misunderstanding of the instructions and, consequently, a failure of the devaluation procedure.

To test this hypothesis, we first conducted an LMM analysis with press frequency as the dependent variable. Phase (pre vs. post-devaluation), cue (valued vs. devalued), and outcome liking score were included as fixed effects and interactions. We used normalized (standardized) measures for both outcome liking and press frequency. To account for interindividual variability, we included trial order, individual intercepts, and subject-specific slopes for the cue × phase interaction as random effects.

The two latent participant clusters were identified based on the behavioral adaptation index (BAI)—that is, the change in behavior before and after devaluation—and were presumed to reflect differences in sensitivity to changes in outcome value. However, if behavioral change largely depends on devaluation efficacy, these clusters may instead reflect differences in participants’ understanding of the devaluation instructions. To examine group-level differences in devaluation efficacy between clusters, we built another linear mixed model (LMM) with outcome liking scores as the dependent variable and the interaction between cue (valued vs. devalued) and cluster (outcome-sensitive vs. outcome-insensitive) as predictors. Due to convergence issues, we included only the individual intercept in the random effects structure.

Finally, we computed a devaluation efficacy index (DEI) by subtracting the liking rating for the valued outcome from that of the devalued outcome (DEI = valued outcome liking − devalued outcome liking). Higher DEI values indicated more effective devaluation; values near the negative extreme suggested misunderstanding (i.e., perceiving the devalued outcome as more attractive), while values near zero indicated participants rated both outcomes similarly post-devaluation (i.e., they missed or ignored the devaluation warnings). After confirming that none of the measures followed a normal distribution, we examined correlations between DEI and BAI using Spearman’s rank correlation coefficient.

**Reanalyses of data from **Pool et al. (2022)** and **Gera et al. (2023)**: Relationship between devaluation efficacy and changes in response rate due to devaluation**. We retrieved the original datasets from Pool et al. (2022) and Gera et al. (2023) from their open-access GitHub repositories (respectively, https://github.com/evapool/MULTILAB_HABIT; https://github.com/ranigera/MultiModalMRI_Habits). We first replicated the main analyses to extract data related to the manipulation checks (i.e., outcome liking and hunger ratings) and the devaluation effects on response rates as reported by the authors. It should be noted that the clustering results differed slightly because Pool et al. (2022) normalized the BAI using the full raw sample, whereas we applied normalization after excluding participants who did not meet the inclusion criteria. Despite this difference, the cluster solution and sample distribution across clusters were nearly identical to those reported in the original study.

We used the changes observed in hunger and pleasantness ratings before and after the devaluation procedure as two markers of the effectiveness of devaluation for each participant. The hunger change index (CHI) was calculated by subtracting the post-devaluation hunger rating from the pre-devaluation rating [hunger change index (HCI) = (hunger rating pre) – (hunger rating post)]. The pleasantness change index (PCI) was calculated by subtracting the pre–post devaluation change in outcome liking scores for the valued cue from the change observed for the devalued cue, following the same logic as the BAI [pleasantness change index (PCI) = (outcome liking post valued – outcome liking pre valued) − (outcome liking post devalued – outcome liking pre devalued)].

Higher PCI and HCI values indicate greater devaluation effectiveness—that is, participants’ hunger decreased, and they rated the devalued snack as less desirable after satiation, particularly relative to the valued snack. Scores around zero would indicate that there were barely any changes in how attractive the snacks were to participants (or changes existed but were identical for both snacks), nor in their hunger after satiation. Negative values indicate either an increase in hunger or pleasantness ratings after devaluation or, in the case of the PCI, that the non-sated (still-valued) food lost more attractiveness than the devalued one.

We hypothesized that changes in response rates were dependent on the efficacy of the satiation procedure. To test this, we included the HCI and PCI as fixed effects in an LMM to determine whether they interacted with the cue and phase factors in explaining changes in response rates. As before, we included individual intercepts as well as the simple main effects of cue and phase in the random effects structure.

We also examined whether the efficacy of devaluation differed between the clusters identified using the BAI, which were assumed to reflect differences in outcome sensitivity. To do this, we ran two additional linear mixed models (LMMs), using cue (valued vs. devalued), phase (pre vs. post), and cluster (outcome-sensitive vs. outcome-insensitive) as predictors of outcome liking and hunger scores. For the model predicting outcome liking scores, the random effects structure included individual intercepts and by-participant random slopes for the main effects of cue and phase. For the model predicting hunger scores, we included only individual intercepts due to convergence issues.

Finally, we computed Spearman’s rank correlations between the HCI, PCI, and BAI measures to assess the associations among these variables directly.

## Results

### Preregistered analyses

#### Manipulation check

**Fig. 2:**
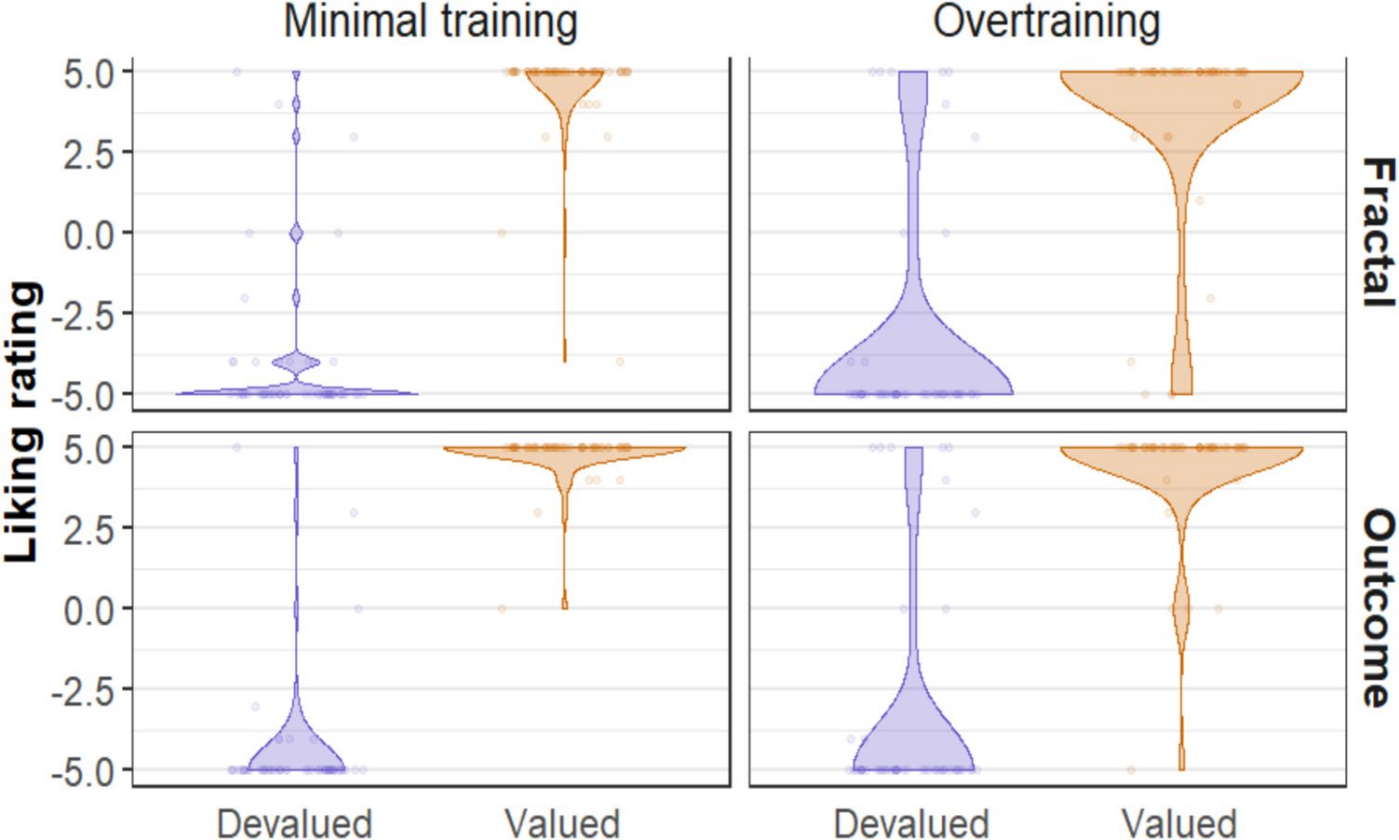
Liking ratio distributions for valued vs. devalued outcomes and fractals. *Note*. The plot displays the pleasantness scores given by each training group for fractals and outcomes after applying devaluation. As shown, ratings for the valued cues are concentrated near the upper end of the scale, indicating high average pleasantness, while ratings for the devalued cues shift toward the negative end of the scale, reflecting reduced pleasantness

Figure 2 presents the results from the 2 × 2 ANOVA analyses. These analyses revealed a significant main effect of cue value for both outcome [*F*(1, 105) = 631.45, *p* < .001, *η*^2^ = .86] and fractal [*F*(1, 105) = 378.52, *p* < .001, *η*^2^ = .78]. Post hoc comparisons showed that pleasantness ratings were significantly lower for the devalued outcome compared to the valued one in both the minimal training condition [*t*(1,105) = −19.46, *SE* = 0.48, *p* <.001, *d* = 3.80] and overtraining condition [*t*(1,105) = −16.13, *SE* = 0.49, *p* < .001, *d* = 3.15]. Similar patterns emerged when evaluating fractal preferences (minimal training: *t*(1,105) = −15.15, *SE* = 0.58, *p* < .001, *d* = 2.96; overtraining: *t*(1,105) = −12.41, *SE* = 0.59, *p* < .001, *d* = 2.42). As expected, since the liking tests were administered after the devaluation instructions, this pattern confirms that participants rated the devalued outcomes and their associated fractals as significantly less pleasant than the valued ones.

There was no main effect of training condition for either outcome [*F*(1, 105) = 1.80, *p* = .182, *η*^2^ = .02] or fractal [*F*(1, 105) = 0.03, *p* = .869, *η*^*2*^ = .00].

Notably, a marginally significant interaction effect emerged between the training group and outcome [*F*(1, 105) = 3.99, *p* = .048, *η*^2^ = .04], likely due to a greater difference in pleasantness scores between the valued and devalued cues in the minimally trained participants. However, post hoc comparisons did not reveal any significant differences between the training groups.

Bayesian analyses provided no evidence supporting an interaction effect between group and cue factors compared to a model including only the fixed effects (*BF*_10_ outcome = 0.36 ± 1.85%; *BF*_10_ fractal = 0.77 ± 1.31%). In fact, the model that included only the cue factor obtained more support than an alternative model that also included the group factor (*BF*_10_ outcome = 4.02 ± 0.75%; *BF*_10_ fractal = 6.17 ± 0.79%). This suggests that cue value was the primary—and almost sole—factor explaining the distribution of the data.

### Outcome devaluation changes

The interaction of cue × group was not significant [*F*(1, 105) = 1.94, *p* = .166, *η*^2^ = .02], indicating that the two training groups did not show differential reductions in responses to devalued trials compared to valued ones (i.e., evidence of outcome sensitivity regardless of the amount of training).

We observed a significant main effect of cue [*F*(1, 105) = 261.33, *p* < .001, *η*^2^ = .71] reflecting higher response rates for valued trials compared to devalued trials^2^ in both the minimal training condition (*t*(1,105) = −10.60, *SE* = 0.30, *p* <. 001, *d* = 2.07) and the overtraining condition (*t*(1,105) = −12.25, *SE* = 0.31, *p* <. 001, *d* = 2.39).

There was also a significant main effect of training condition [*F*(1, 105) = 6.97, *p* = .010, *η*^2^ = .06], indicating that the overtraining group responded more frequently overall than the minimal training group. However, post hoc paired comparisons did not reveal significant differences between training conditions for either the devalued cue (*t*(1,105) = −2.10, *SE* = 0.39, *p* = .161, *d* = 0.41) or the valued cue (*t*(1,105) = 1.46, *SE* = 0.14, *p* = .466, *d* = 0.28).

**Fig. 3:**
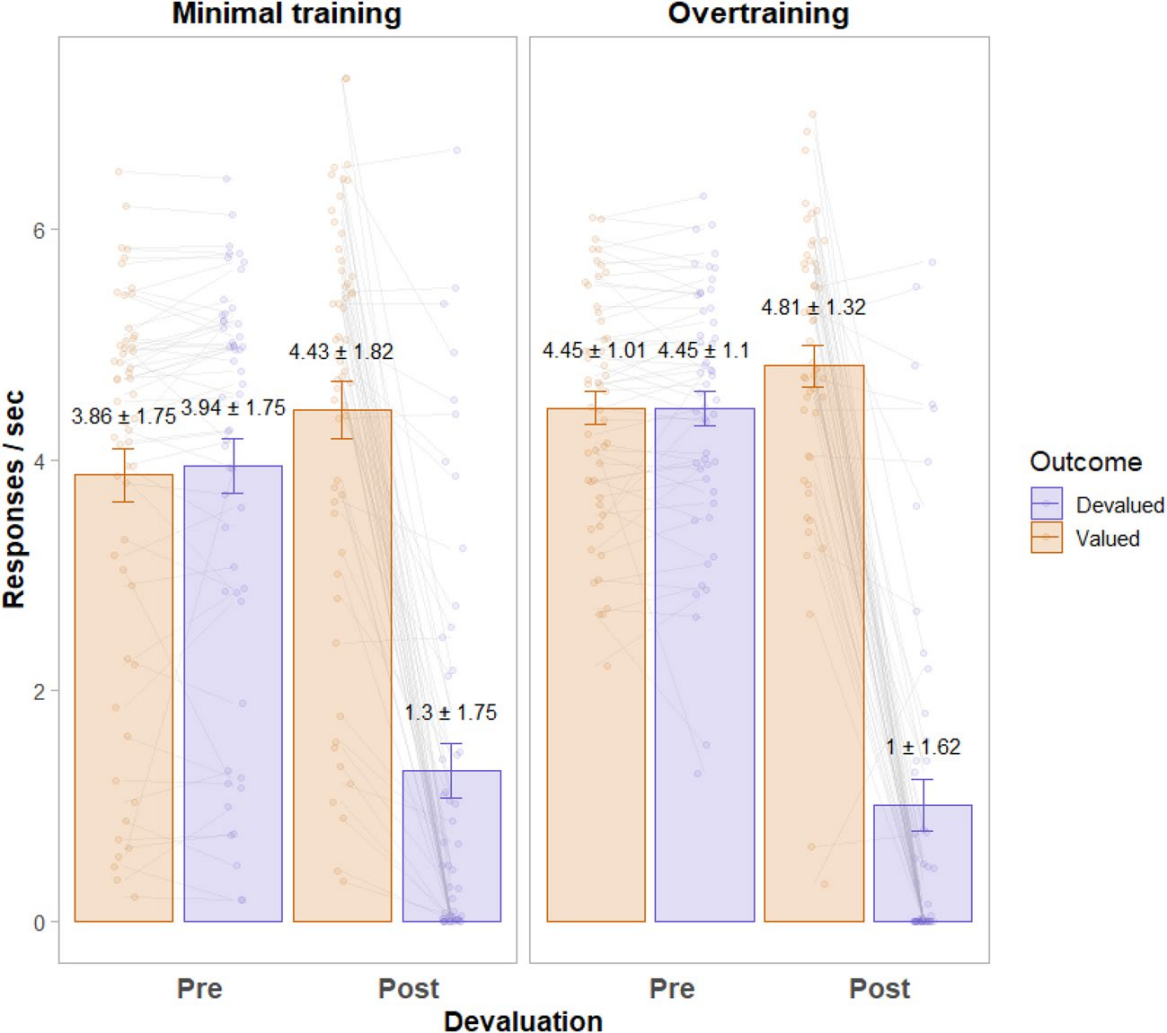
Response rates for valued and devalued outcomes before and after devaluation. *Note*. Mean press frequencies for valued and devalued outcomes, before and after devaluation, are shown separately by training condition. The response patterns were similar across training groups: equivalent response rates for both valued and devalued outcomes prior to devaluation, followed by a reduction in responses to the devalued outcome post-devaluation, while responses to the valued outcome remained high. Notably, the overtraining group exhibited higher overall response frequencies across all conditions—except for the devalued outcome after devaluation—compared to the minimal training group

Bayesian analyses supported the same conclusion: the model including only the main effects of group and cue showed stronger evidence than the alternative model that included the interaction effect (*BF*_10_ = 1.62 ± 3.70%). Figure 3 shows the mean response rates for each combination of factors.

### Cluster analyses

**Fig. 4:**
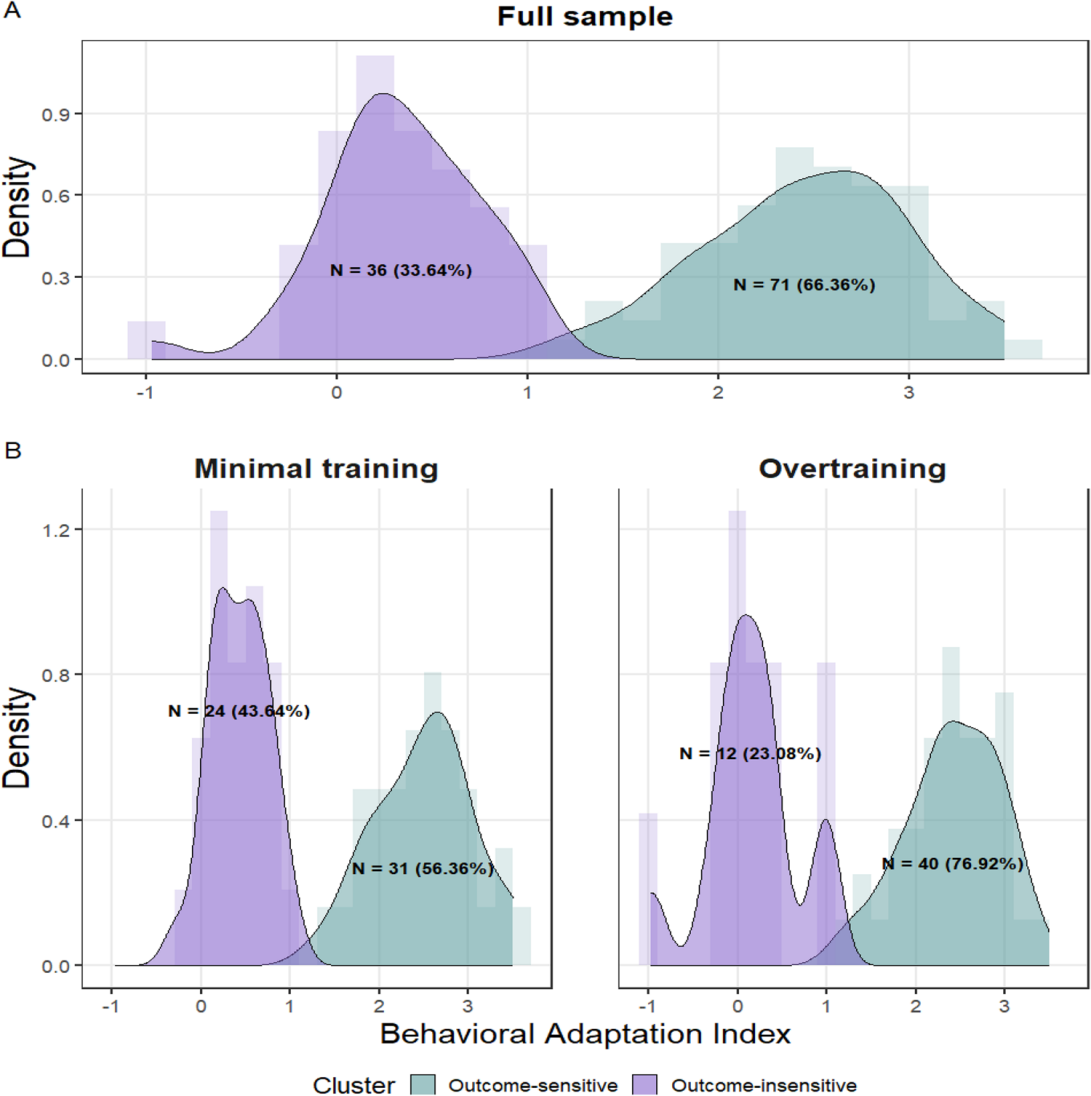
Density chart for cluster solution based on the behavioral adaptation index. *Note*. **A** Graphical representation of the best cluster solution for behavioral adaptation index (BAI) across the whole sample (*N* = 107). **B** Distribution of outcome-sensitive and outcome-insensitive participants separated by training condition. The majority of participants were outcome-sensitive in both the minimal training group (*N* = 31) and the overtraining group (*N* = 40), indicating that most participants substantially adjusted their behavior in response to the changes in outcome value imposed by devaluation

The statistics for each clustering model are displayed in Table 2. Based on the BIC index, the best-fitting solution identified two latent clusters (Fig. 4A). One cluster included participants whose behavioral adaptation index (BAI) scores showed a positive tendency (*N*₁ = 36), but the values were closer to zero. This pattern suggests that participants in this cluster showed minimal modulation of their responses following the devaluation procedure, a profile more consistent with habitual control. For consistency with prior studies (Gera et al., 2023; Pool et al., 2022), we labeled this cluster as *outcome-insensitive*.

**Table 2:** Model fit statistics for cluster solutions

*k*	logLik	AIC	BIC	Cluster sizes
1	−162.53	331.05	339.07	107
**2**	−**139.16**	**292.31**	**311.02**	**71; 36**
3	−133.14	288.27	317.67	51; 37; 19
4	−129.77	289.54	329.64	45; 33; 21; 8
5	−126.34	290.67	341.46	35; 32; 21; 10; 9

*k* = number of clusters; logLik = log likelihood; BIC = Bayesian information criterion

However, as shown in Fig 4A, participants in this first cluster were not completely outcome-insensitive, as their BAI values were mostly distributed above zero. The second cluster (*N*₂ = 71) showed BAI values that were more clearly shifted toward positive values, which is why we labeled this group as outcome-sensitive. Notably, the outcome-sensitive cluster comprised the majority of the sample, nearly double the number of participants compared to the outcome-insensitive cluster.

We then compared the proportion of participants assigned to each cluster across the minimal training and overtraining conditions (Fig. 4B). The chi-squared test revealed a significant difference in cluster distribution between the two groups [*χ*^2^(1) = 4.18; *p* = 0.041]. Specifically, the proportion of outcome-sensitive participants was higher in the overtraining group compared with the minimal training group. Nonetheless, even in the minimal training condition, the outcome-insensitive cluster represented only a minority of participants.

### Analysis of the moderating effects of individual differences

**Fig. 5:**
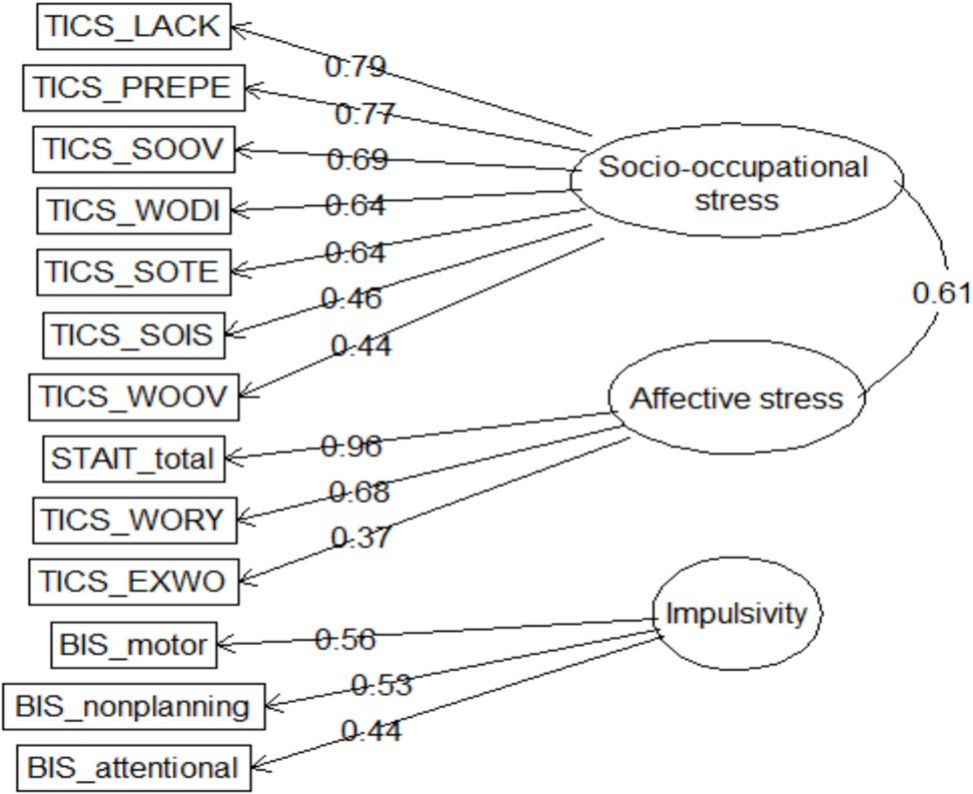
Factorial solution for measures of individual differences. *Note*. Results revealed three main latent factors, named *socio-occupational stress*, *affective stress*, and *impulsivity*.

The EFA supported a three-factor model (Fig. 5). The first factor, which we labeled *impulsivity*, was composed solely of the dimensions from the Barratt Impulsiveness Scale. The second factor, *socio-occupational stress*, included several subscales from the Trier Inventory for Chronic Stress (TICS). The third factor, labeled *affective stress*, was primarily correlated with the STAI-Trait score and also included two TICS dimensions: chronic worrying (TICS_WORY) and excessive demands at work (TICS_EXWO). The validity coefficients were *R*^2^₍_Imp_₎ = 0.82, *R*^2^₍_Socio-occup_₎ = 0.95, and *R*^2^₍_AffectStress_₎ = 0.96 (see Supplemental Material B1 for full subscale factor loadings).

When naming these factors, we aimed to follow the terminology used by Pool et al. (2022) to facilitate cross-study comparisons. However, our results diverged notably from theirs, and the composition of the affective stress dimension differed between studies, meaning it cannot be directly equated. Interestingly, our factorial structure closely resembled that reported by Gera et al. (2023). We also found that affective stress and socio-occupational stress shared a moderate degree of common variance, as reflected by their significant correlation (*r*^2^ = 0.61, *p* < .001), which is theoretically consistent given that both factors capture stress-related conditions and symptoms.

We conducted an LMM separately for each factor (see Supplemental Material B2 for details). None of the factors showed significant interaction effects, indicating that they did not modulate the response changes before and after devaluation across training groups (socio-occupational stress: *β* = 0.18, *SE* = 0.22, 95% CI [−0.25, 0.61], *p* = .41; affective stress: *β* = 0.18, *SE* = 0.22, 95% CI [−0.24, 0.60], *p* = .40; impulsivity: *β* = 0.16, *SE* = 0.22, 95% CI [−0.28, 0.59], *p* = .48).

Following the strategy of Pool et al. (2022), we repeated the EFA using the *varimax* orthogonal rotation method. This yielded a slightly different solution, with the TICS_EXWO subscale (previously part of the *affective stress* factor) now loading onto *socio-occupational stress*. As this factorial structure aligned better with theoretical expectations, we reran the LMM using this solution. However, once again, we found no significant interaction effects (socio-occupational stress: *β* = 0.13, *SE* = 0.22, 95% CI [−0.30, 0.56], *p* = .56; affective stress: *β* = 0.13, *SE* = 0.22, 95% CI [−0.29, 0.55], *p* = .54; *impulsivity*: *β* = 0.15, *SE* = 0.22, 95% CI [−0.28, 0.58], *p* = .50).

## Exploratory analyses

### Relationship between devaluation efficacy and changes in response rate following devaluation

Our LMM revealed a significant interaction between cue, phase, and outcome liking (*β* = 0.53, *SE* = 0.18, 95% CI [0.18, 0.88], *p* = .003), indicating that pre–post devaluation changes in press frequency for each cue were influenced by pleasantness ratings—that is, by devaluation efficacy.

When examining the participant clusters identified through the BAI, we found a significant interaction between cue and cluster (*β* = 0.44, *SE* = 0.04, 95% CI [0.37, 0.51], *p* < .001). Differences in pleasantness ratings between valued and devalued outcomes varied across clusters (see Supplemental Material B4 for detailed estimates of main effects and interactions).

**Fig. 6:**
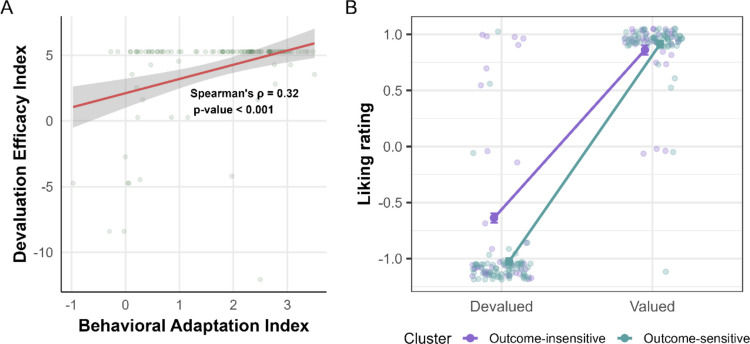
Relationship between devaluation efficacy and behavioral change. *Note.*** A** Graphic representation of BAI and DEI (DEI = outcome valued liking rate – outcome devalued liking rate). The two measures were significantly and positively correlated (ρ = 0.32, *p* < .001). Thus, the participants for whom the devaluation was most effective were also those who most strongly adapted their responses to changes in outcome value following the devaluation instructions. **B** Liking ratings for valued and devalued outcomes in each participant cluster. While both clusters rated the still-valued outcome as more attractive than the devalued one, the outcome-insensitive participants gave significantly higher liking scores for the devalued outcome compared to the outcome-sensitive participants

Post hoc comparisons showed that, although both clusters rated the valued outcome as more pleasant than the devalued outcome (outcome-insensitive: *t*(1,1821) = −52.04, *SE* = 0.03, *p* < .001, *d* = 2.44; outcome-sensitive: *t*(1,1821) = −94.48, *SE* = 0.02, *p* < .001, *d* = 4.43), participants in the outcome-insensitive cluster rated the devalued outcome as significantly more pleasant compared to those in the outcome-sensitive cluster (*t*(1, 141) = 7.58, *SE* = 0.05, *p* < .001, *d* = 1.28) (Fig. 6B). No significant difference was found between clusters in liking scores for the valued outcome (*t*(1, 141) = −1.00, *SE* = 0.05, *p* = .320, *d* = 0.17). All multiple post hoc comparisons remained significant after applying Tukey’s HSD correction. Thus, the participants who showed more habit-like behavior were also those who tended to rate the devalued outcome as more appealing (raising rational doubts about whether they were actually pursuing that reward).

Finally, we found a positive and significant correlation between the behavioral adaptation index (BAI) and the pre-calculated devaluation efficacy index (DEI) (ρ = 0.32, *p* < .001). This indicates that the more effective the devaluation had been, the larger the pre–post change in response frequency (Fig. 6A).

### Reanalyses of data from Pool et al. (2022) and Gera et al. (2023): Relationship between devaluation efficacy and changes in response rate due to devaluation

The LMM analyses did not reveal a significant interaction between cue, phase, and the hunger change index (HCI) on press frequency, in either the Pool dataset (*β* = 0.01, *SE* = 0.04, 95% CI [−0.06, 0.08], *p* = .746) or the Gera dataset (*β* = 0.04, *SE* = 0.06, 95% CI [−0.08, 0.16], *p* = .501). Thus, the change in hunger levels does not seem to influence the response rates for each cue due to the devaluation procedure.

In contrast, the pleasantness change index (PCI) showed a significant interaction with cue and phase factors on both the Pool (*β* = −0.06, *SE* = 0.03, 95% CI [−0.12, −0.001], *p* = .045) and Gera datasets (*β* = −0.33, *SE* = 0.05, 95% CI [−0.43, −0.23], *p* < .001). This indicates that the differences in response rates for each cue before and after devaluation were modulated by the PCI score, which reflects participants’ proper understanding of the devaluation instructions and, consequently, the efficacy of devaluation. Detailed estimates of all factors and interactions are reported in Supplemental Material C1.

We further explored differences in devaluation efficacy between the previously identified participant clusters. For hunger levels, we found a significant phase × cluster interaction in both the Pool dataset (*β* = 0.16, *SE* = 0.03, 95% CI [0.11, −0.22], *p* <..001) and the Gera dataset (*β* = 0.26, *SE* = 0.04, 95% CI [0.18, 0.34], *p* < .001). However, post hoc comparisons between clusters did not reach significance, either before devaluation (Pool dataset: *t*(1, 320) = −0.73, *SE* = 0.07, *p* = .467, *d* = 0.08; Gera dataset: *t*(1, 128) = 0.32, *SE* = 0.13, *p* = .747, *d* = 0.06) or after devaluation (Pool dataset: *t*(1, 352) = 1.64, *SE* = 0.07, *p* = .101, *d* = 0.18; Gera dataset: *t*(1, 137) = −1.68, *SE* = 0.13, *p* = .095, *d* = 0.29). The decrease in hunger levels following devaluation was significant in both datasets, even after applying Tukey’s HSD correction, and was observed in both the outcome-insensitive cluster (Pool dataset: *t*(1, 4,953) = −100.85, *SE* = 0.02, *p* <..001, *d* = 2.87; Gera dataset: *t*(1, 2,093) = 49.67, *SE* = 0.03, *p* < .001, *d* = 2.17) and the outcome-sensitive cluster (Pool et al. dataset: *t*(1, 4,953) = −73.18, *SE* = 0.02, *p* < .001, *d* = 2.08; Gera dataset: *t*(1, 2,093) = 32.80, *SE* = 0.03, *p* < .001, *d* = 1.43).

**Fig. 7:**
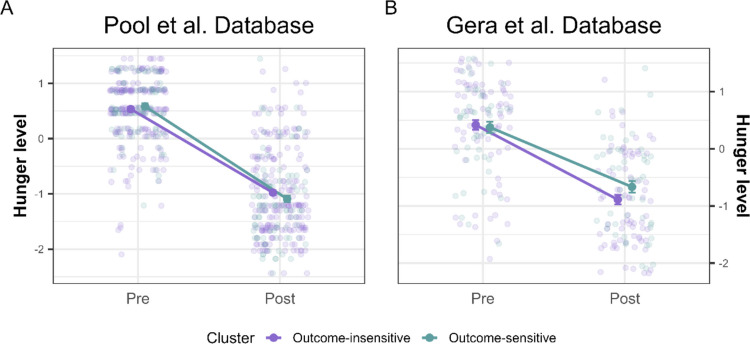
Reported hunger levels by participant clusters before and after devaluation. *Note*. Reported hunger levels by participant clusters before and after devaluation in (**A**) the Pool et al. (2022) dataset and (**B**) the Gera et al. (2023) dataset. In both clusters across both datasets, participants’ hunger significantly decreased following the satiation procedure. Although a phase × cluster interaction emerged, post hoc comparisons did not reveal any significant differences in hunger levels between clusters, either before or after devaluation

In the Pool et al. (2022) dataset, the phase × cluster interaction appeared to be driven by a smaller decrease in hunger levels within the outcome-insensitive cluster compared with the outcome-sensitive cluster (Fig. 7A). In contrast, the Gera et al. (2023) dataset showed the opposite pattern (Fig. 7B). However, it is important to note that the hunger change index (HCI) did not interact with direct measures of response rate in the earlier LMM analyses, nor were there significant between-group differences in the post hoc comparisons. Therefore, we believe the observed interaction in this analysis may be an artifact, likely arising from the use of the clustering variable (derived from press frequency) rather than reflecting a genuine association between HCI and the response pattern underlying cluster assignment. Consistent with this interpretation, subsequent analyses revealed no significant correlation between HCI and BAI variables in either the Pool dataset (ρ = 0.049, *p* = .785) or the Gera dataset (ρ = −0.164, *p* = .138) (see Fig. 9B). All *p*-values were reported using Tukey’s HSD correction.

**Fig. 8:**
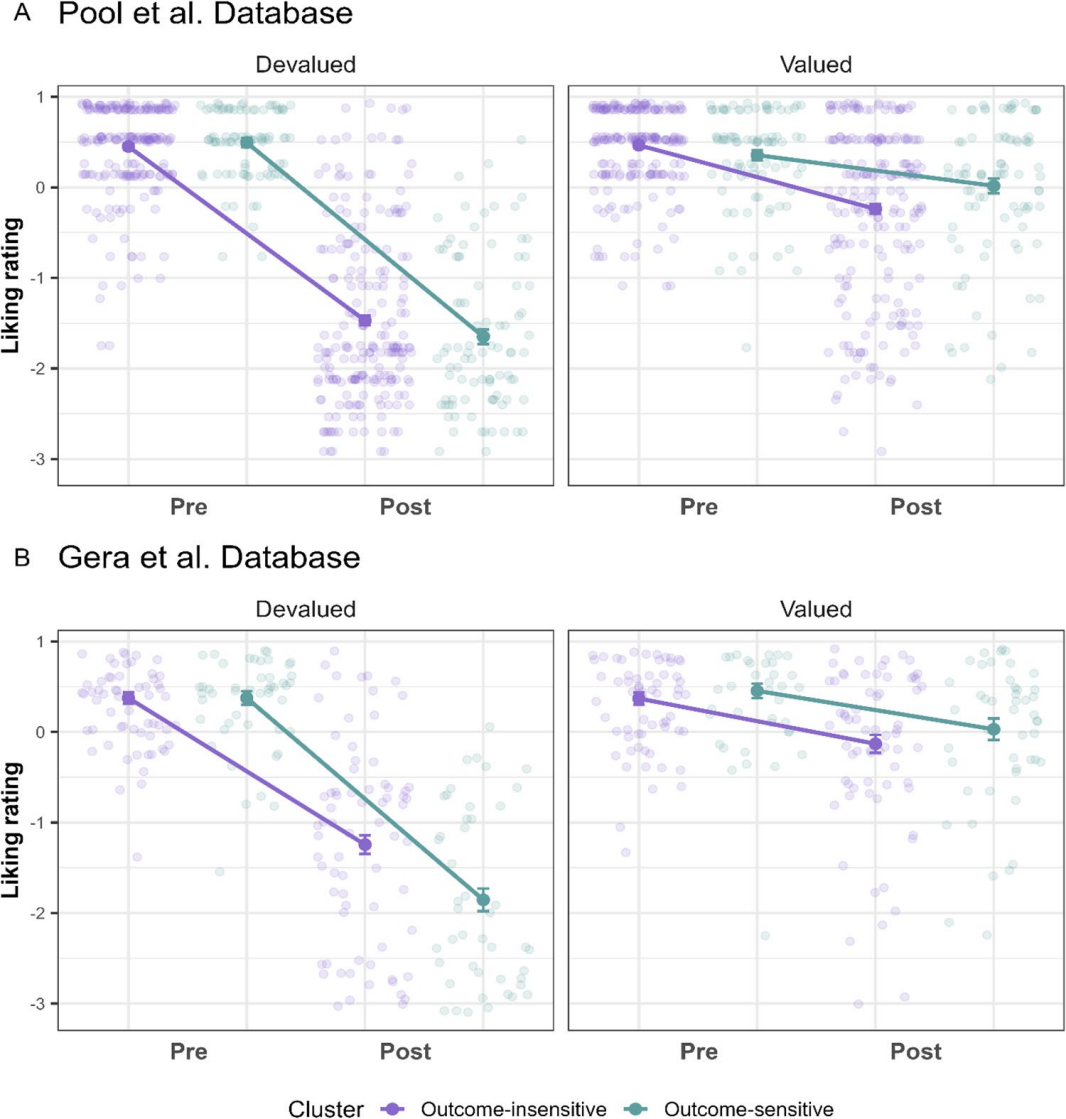
Reported liking ratings for outcomes by participant clusters before and after devaluation. *Note.* The drop in pleasantness ratings for valued and devalued outcomes differed significantly between participant clusters in both (**A**) the Pool et al. (2022) dataset and (**B**) the Gera et al. (2023) dataset. Outcome-sensitive participants show a marked decrease in liking scores for the devalued outcome compared to the outcome-insensitive cluster. Conversely, for the valued outcome, the decrease was more modest in the outcome-sensitive group, while the outcome-insensitive group showed a steeper decline, at least in the Pool dataset.

Regarding pleasantness scores, we found a significant triple interaction between phase, cue, and cluster in both datasets. In the Pool dataset, this triple interaction effect (*β* = 0.59, *SE* = 0.04, 95% CI [0.51, 0.67], *p* < .001) was likely due to a larger decrease in pleasantness ratings for the valued cue from pre- to post-devaluation among outcome-insensitive participants (Fig. 8A). Post hoc comparisons revealed that after devaluation, the outcome-insensitive cluster showed significantly lower liking ratings for the valued cue compared to the outcome-sensitive cluster (*t*(1, 320) = −2.63, *SE* = 0.10, *p* = .009, *d* = 0.29). However, this difference did not remain significant after applying Tukey’s HSD correction.

In the Gera et al. database, the phase × cue × cluster interaction (*β* = 0.69, *SE* = 0.05, 95% CI [0.58, 0.79], *p* < .001) was driven by the outcome-insensitive group rating the devalued cue as significantly more pleasant than the outcome-sensitive group after devaluation (*t*(1, 128) = 3.77, *SE* = 0.16, *p* < .001, *d* = 0.67) (Fig. 8B). This difference remained significant even after applying Tukey’s HSD correction. Detailed post hoc comparison results for both datasets, using various correction methods, are reported in the Supplemental Material (C2).

**Fig. 9:**
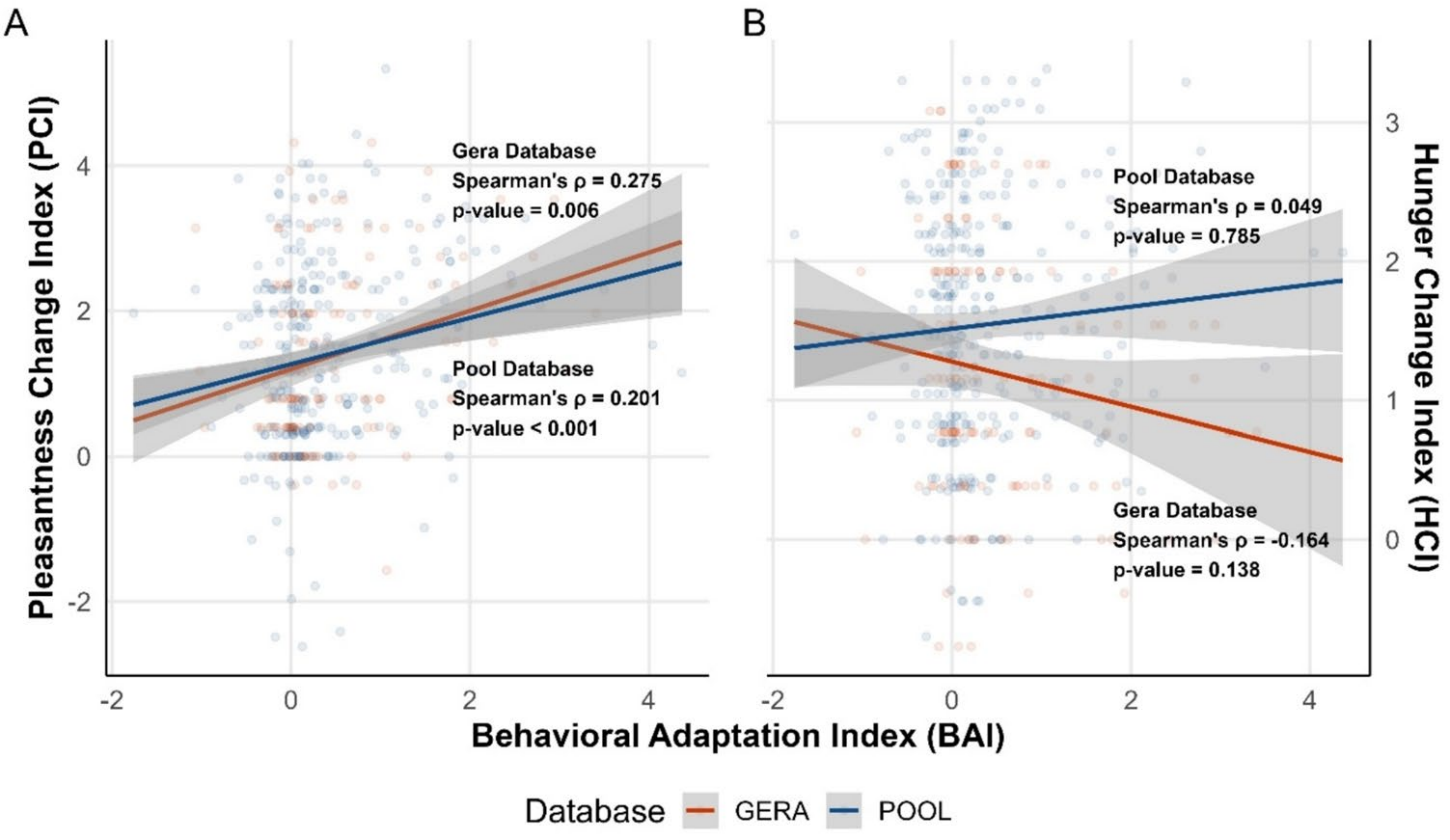
Correlation analyses between devaluation efficacy indexes and the BAI. *Note.*** A** Correlation analyses between the BAI and PCI scores revealed a significant positive association between these variables across both datasets. This indicates that the larger the pre–post devaluation changes in pleasantness scores (i.e., the more effective the devaluation procedure), the greater the behavioral adaptation displayed by participants. **B** No significant correlation was found between the BAI and HCI scores in either dataset

Finally, Spearman’s rank coefficient analyses revealed a significant positive correlation between the behavioral adaptation index (BAI) and the corresponding *pleasantness change ind*ex (PCI) in both the Pool dataset (ρ = 0.201, *p* < .001) and the Gera dataset (ρ = 0.275, *p* = .006). This indicates that lower PCI scores were associated with less behavioral change (Fig. 9A). In contrast, the pre–post difference in hunger levels showed no significant correlation with BAI in either dataset (Fig. 9B).

## Discussion

The study by Tricomi et al. (2009) was groundbreaking as it was the first to demonstrate an increase in habitual responding due to overtraining in a human laboratory setting. However, several subsequent attempts to replicate these crucial findings have failed, showing similar levels of habit expression regardless of the amount of prior instrumental training (de Wit et al., 2018; Gera et al., 2023; Pool et al., 2022). Recently, Pool et al. (2022) and Gera et al. (2023) proposed that this null effect was due to most participants reaching a ceiling level of habitualization even under minimal training. This maximal level of habitual responding, even in conditions with less training, would impede the detection of statistical differences compared with long-trained conditions. Alternatively, we suggest that apparent habitual responses observed in this protocol (regardless of the amount of training) were indeed an artifact resulting from shortcomings in the devaluation protocol.

We preregistered a conceptual replication of Pool et al. (2022), adapting Tricomi’s devaluation procedure by replacing food rewards with monetary rewards, aiming to overcome several limitations associated with food-based paradigms (e.g., inability to compel consumption, social stigma around overeating, and confusion about the task’s ultimate goal). Similar to prior work by Gera et al. (2023) and Pool et al. (2022)—and in contrast to Tricomi’s original findings—we found no differences in habitual responding between the minimal training and overtraining conditions. This result suggests that this task could not be appropriate for studying habit acquisition, at least in humans (see Watson et al., 2022).

However, previous research employed cluster analyses to argue that the null result of overtraining occurred because a majority of participants exhibited habitual (i.e., outcome-insensitive) behavior even under minimal training (Gera et al., 2023; Pool et al., 2022). Importantly, as we expected, this explanation does not apply to our data. In our sample, participants who remained goal-directed after devaluation made up the vast majority (66.36%), forming the *outcome-sensitive* cluster. Moreover, even those classified as outcome-insensitive in our study showed a great degree of sensitivity to outcome devaluation (Fig. 4A).

This predominance of outcome-sensitive participants was maintained across both training conditions. In fact, the proportion of outcome-insensitive participants was smaller in the overtrained group compared to the minimal training group. That is, overtraining appeared to favor goal-directed processes, likely because participants in this condition were more familiar with the task mechanics and less confused by the devaluation instructions. These findings challenge the argument that the free-operant task induces habits after just one session, thereby capping habit levels and eliminating differences between minimal and extended training (Gera et al., 2023; Pool et al., 2022). Not only did we observe a low rate of habit-like responses in the minimal training group, but participants actually appeared to become *less* habitual as training increased. This pattern is difficult to reconcile with the ceiling hypothesis proposed by Gera et al. (2023) and Pool et al. (2022).

It is worth noting that, theoretically, behavior should switch from goal-directed to habitual control as a function of repeated experience with S-R-O associations (Buabang et al., 2025). Therefore, any measure intended to be a valid index of habit strength must be able to track the progressive habitualization of behavior with increasing instrumental training (Luque et al., 2020; Martínez-López et al., 2026).

Why did we fail to observe between-group differences in habitual responses, with our participants remaining mostly goal-directed regardless of the amount of instrumental training? According to the associative dual-process framework for instrumental behavior, goal-directed control relies on R-O (response–outcome) representations to select the optimal action for achieving a desired outcome, whereas habitual behavior is triggered almost automatically by specific cues, based on past S-R associations (Watson et al., 2022). The evolutionary advantage of this stimulus-driven system lies in its speed and low cognitive cost, enabling rapid actions that were often beneficial in stable, everyday contexts. Nonetheless, if R-O contingencies change and habits are no longer aligned with current goals (as in the outcome devaluation test), a conflict arises. Recent research has shown that when individuals have sufficient time and available resources, cognitive control can effectively inhibit now-inappropriate habits and favor goal-directed behavior (Buabang et al., 2025; Hardwick et al., 2019; Littman et al., 2019; Luque et al., 2020).

In this line, recent findings strongly suggest that limiting the time available to respond is a key factor for successfully measuring the behavioral expression of habits (Hardwick et al., 2019; Luque et al., 2020). Hardwick et al. (2019) designed a task in which participants learned four different S-R associations, either in a single session or over four consecutive days of practice. In the test phase, two of the trained stimuli were switched to require a different motor response. These authors found that habitual errors were significantly more frequent following overtraining than following minimal training, but crucially, this effect emerged only on trials in which participants had little time to prepare their response after stimulus onset. When longer response preparation times were allowed, the effect of training disappeared. Similarly, Martínez-López et al. (2026) reported that even when participants successfully adjusted their responses to new S-R contingencies, those in the overtraining group required more time to produce the correct, goal-directed action compared to the minimal training group. These results align with those of Luque et al. (2020), who observed greater slowing (reaction-time switch costs) when participants responded correctly to devalued versus still-valued outcomes. Notably, these switch costs increased with training but were only detectable under time pressure conditions (see also Nebe et al., 2024).

Considering these findings, since the outcome devaluation test in the free-operant task is self-paced, it gives participants the time and cognitive space to resolve this conflict in favor of their current goals. Therefore, current findings suggest that if habits were formed during training in Tricomi’s task (we cannot know if that was the case) and the devaluation procedure was perfect (we do know that this was not the case), the self-paced nature of the task (e.g., no time constraints on responding) would allow the goal-directed system to override possible habitual responses during the test.

Another common feature of tasks that have shown sensitivity to overtraining, such as those used by Hardwick et al. (2019) and Luque et al. (2020), is that participants were not merely asked to inhibit an action, as in the free-operant paradigm, but were required to make a motor response that was critically different from the one they had previously learned. In a similar vein, Du and Haith (2025) found that the process of selecting and preparing a response, which is crucial for tasks requiring remapping of previous S-R associations, is more prone to *habitualization* than the movement initiation process itself. The latter, they argue, is the central component of withholding paradigms, in which participants are not asked to restructure their responses but simply to refrain from acting. Du and Haith’s (2025) hypothesis, if true, would imply another reason to remain skeptical about the appropriateness of Tricomi’s task for studying habit formation, because the free-operant task requires withholding the habitual response during the test of habits. In summary, the null differences between training conditions, both in our dataset and in previous studies, may be attributed to task features that impede the capture of overt habitual responses due to goal-directed overriding processes.

Given this body of evidence, it is somewhat surprising that in the Pool et al. (2022) and Gera et al. (2023) experiments, the majority of participants continued responding to the devalued outcome during a self-paced test (with no response time pressure). Our hypothesis was that these seemingly “habitual” participants were, in fact, acting under goal-directed control. Their response patterns resembled outcome insensitivity (habitual behavior) because the supposedly *devalued outcome* had not been effectively devalued (i.e., it retained its motivational power even after selective satiation), so participants continued to desire the reward and acted accordingly. To explore this hypothesis, we analyzed the relationship between devaluation effectiveness and habit-like responses.

The current results support this idea. Across all three examined datasets, behavioral changes from pre- to post-devaluation were conditioned by how attractive participants still found the rewards, regardless of the amount of training. Those who continued to rate the devalued outcome in high regard were also the ones who showed the least change in response patterns after devaluation, across both outcomes.

In the Gera et al. (2023) study, the cluster classified as outcome-insensitive rated the devalued outcome as significantly more appealing than the outcome-sensitive cluster, similar to what we observed in our dataset. We argue that these participants were still actively pursuing the outcome during the test phase. The limitations of devaluation paradigms based on selective satiation for investigating habits in humans have already been drawn by other authors (Eder & Dignath, 2019; Smeets et al., 2023). For instance, Buabang et al. (2023) conducted a conceptual replication of the classic study by Schwabe and Wolf (2011), which reported a greater tendency to behave in an outcome-insensitive manner under stress after applying selective satiation. In contrast, Buabang et al. (2023) used taste aversion for devaluation and found that both stressed and non-stressed participants rejected the devalued outcome. The authors suggested these discrepancies could be due to the weaker effect of selective satiation in the original study. Similarly, Hogarth and Chase (2011) attempted to devalue tobacco through specific satiation in a sample of smokers using a *Pavlovian instrumental transfer* paradigm. Even after devaluation, participants continued to respond at high rates to cues associated with tobacco in the first conditioning phase (i.e., S_1_-O_1_). Smokers were perfectly aware that the state of satiety would be temporary, so they kept the goal of acquiring more cigarettes to consume later. Similarly, research has shown that the effectiveness of these devaluation paradigms declines when the outcome is not delivered immediately after task completion, and participants are allowed to take it home (Eder & Dignath, 2016), as was the case in the original Tricomi et al. (2009) protocol.

Examining the data from Pool et al. (2022), the devaluation appeared to be less selective for the outcome-insensitive cluster, as they rated the valued outcome as less pleasant compared to their outcome-sensitive counterparts. As a result, their response rates to the devalued outcome emulated those for the valued outcome, not necessarily because they were acting habitually, but because the valued outcome had also become less attractive. The lack of specificity is another potential limitation of satiation-based devaluation. It can equally reduce the motivational value of both outcomes, leading to indiscriminate responding to both valued and devalued cues, which mimics an outcome-insensitive pattern (Smeets et al., 2023) and potentially leads to misinterpretation of the behavior as habitual. Supporting this concern, in both the Pool et al. (2022) and Gera et al. (2023) studies, liking scores for both still-valued and devalued rewards dropped significantly after devaluation (see Supplemental Material C2), suggesting a nonspecific satiation effect.

Taken together, this evidence raises questions about the reliability of selective satiation and free-operant procedures for studying human habits. Rather than capturing habit-controlled behavior, these paradigms may instead reflect goal-directed actions shaped by incomplete or ineffective devaluation, where participants continue to actively pursue the supposedly devalued outcome (Buabang et al., 2021). In our study, by adapting the devaluation procedure, we not only failed to observe an effect of training on response rates but also found that the participants labeled as outcome-insensitive became a minority. In addition, the BAI scores of our outcome-insensitive cluster were not centered around zero (Fig. 4A), as observed in Pool et al. (2022) and Gera et al. (2023); instead, they shifted considerably toward positive values. In fact, the mean BAI score of our so-called outcome-insensitive cluster was comparable to the outcome-sensitive group in prior replications. For this reason, we question the appropriateness of the *outcome-insensitive* label in our context, as these participants still showed a measurable degree of response adaptation (with one third scoring BAI > 0.5). We believe it would be more accurate in our case to distinguish between a *high* and a *low* outcome-sensitive cluster.

In summary, cluster results from previous research, which show a high proportion of participants not adjusting their behavior after devaluation, are likely due to issues with the selective satiation procedure. They did not exhibit outcome insensitivity due to habit formation; rather, the selective satiation procedure did not work as expected, and they continued to pursue the food. Therefore, when using an alternative, more precise devaluation method, most participants had a clearer understanding that one of the previous outcomes had become undesirable and, consequently, stopped responding to obtain it. That is why we found fewer participants labeled as outcome-insensitive, compared to previous replications (and those labeled as outcome-insensitive did show some level of sensitivity to outcome level; see Fig. 4). Notwithstanding, our devaluation protocol showed some variability, and some subjects were confused about devaluation instructions and exhibited habit-like responses, as observed in the studies by Pool et al. (2022) and Gera et al. (2023). Importantly, these perseverant responses are most likely not the result of the habit system but rather goal-directed.

Taking all this into account, we strongly recommend using devaluation procedures that ensure the intended devalued outcome truly acquires an aversive or negative value. Using monetary rewards that are converted into losses after devaluation effectively fulfills this requirement. Additionally, our analyses highlight the importance of assessing devaluation effects at the individual level, rather than relying solely on group-level analyses. This is essential to avoid biased conclusions due to participants who misunderstood the instructions or for whom the devaluation was ineffective. Indeed, some of the most convincing demonstrations of habitual behavior have included explicit criteria to ensure participants understood the contingency changes (Hardwick et al., 2019; Frölich et al., 2023). These studies examined whether the manipulation worked on a subject-by-subject basis, often by setting minimum performance thresholds before the test phase and/or excluding participants who clearly failed to grasp the test mechanics (Martínez-López et al., 2026).

In this context, we identify a key methodological issue in the clustering approach originally proposed by Pool et al. (2022), which was based on a differential score—the behavioral adaptation index (BAI)—calculated from the pre–post devaluation press frequency for each cue. Specifically, the sample was divided into two groups based on the BAI distribution: those with low BAI scores around zero (labeled as outcome-insensitive) and those with high BAI scores (labeled as outcome-sensitive). However, upon revisiting our data, we found paradoxical cases where participants who *never* pressed to obtain the devalued outcome during the test phase were still classified as outcome-insensitive. This misclassification primarily stemmed from the very low baseline response rates to both cues. Although their press frequency decreased after devaluation (sometimes to the minimum possible level), their BAI remained close to zero since all the original response counts used to compute it were near zero. As a result, these participants were wrongly categorized as outcome-insensitive when, in fact, they were clearly not.

We found no moderating effect of individual differences, measured through a battery of questionnaires, on participants’ response patterns. Consistent with the findings of Gera et al. (2023), our data provided clearer evidence supporting the null hypothesis. The broader literature offers mixed results from investigations examining the role of impulsivity (Dietrich et al., 2016; Hogarth et al., 2012; Hinojosa-Aguado & Gonzalez, 2020), chronic stress (Gillan et al., 2021), and sustained negative affect (Snorrason et al., 2016; Patzelt et al., 2019) in shaping proneness toward habitual or stimulus-driven strategies. However, we believe it is essential to exercise caution when interpreting or comparing the results of the studies using the paradigm, as we adapted it from previous work. Given the limitations discussed earlier, we cannot confidently assume that this test effectively captured the functioning of the habit system. In our view, future research on individual differences in habit formation or expression should first ensure that the tools used to measure habitual control demonstrate strong validity and reliability (Martínez-López et al., 2026).

The present research challenges the conclusions drawn by Tricomi et al. (2009). Until the development of more recent protocols (Hardwick et al., 2019; Luque et al., 2020), Tricomi’s paradigm was the first to demonstrate an influence of training on human habit acquisition. This work has inspired numerous subsequent studies, including the direct replications discussed here (Gera et al., 2023; Pool et al., 2022), and has been frequently cited to support hypotheses that, in light of current evidence, should now be treated with caution. For instance, the experimental series conducted by Nebe et al. (2024) included a replication of this same paradigm but omitted the overtraining condition under the assumption that the task reliably generated habits after just 1 day of training.

This study also has some limitations. Including a control group that followed the original selective satiation devaluation procedure would have allowed for more direct comparisons between protocols. Moreover, the fact that the nature of the rewards (monetary vs. food) differed between our study and prior replications introduces a constraint on straightforward comparisons, though this was a necessary trade-off given our research goals. In this vein, some could argue that the lack of outcome insensitivity in our experiment, compared to previous replicas, is due to intrinsic differences in the devaluation procedure. In the case of satiation, participants are expected to experience a direct loss of the outcome value; whereas our paradigm involves devaluation through instructions, which could favor goal-directed processes. However, as we commented above, the main tasks that have captured an increase in habit strength due to training also employed explicit instructions to implement devaluation (Luque et al., 2020; Hardwick et al., 2019). Thus, the mere use of instructions cannot explain the strong tendency towards goal-directed behavior in our sample.

Another limitation is that we did not ask participants to rate the pleasantness of each outcome before devaluation, as in prior studies, which would have facilitated more precise comparisons and enabled us to measure participants' initial motivation through the monetary rewards. Nonetheless, we can reasonably assume that participants were highly motivated to earn money, given the high liking rates of the still-valued outcome after devaluation. Additionally, we provided course credits for participating in the study, which were guaranteed upon completion of all training days, acting as an immediate motivational driver. Importantly, by applying a performance-based filter using the contingency test, we reduced potential noise from participants who failed to correctly learn the associations and may have devalued an outcome simply due to misunderstanding. Finally, the generalizability of our findings is limited by the fact that our sample consisted exclusively of undergraduate students.

The current study offers several recommendations for future research on human habits. First, the success of the devaluation protocol should be carefully assessed, and individual devaluation levels should be explicitly incorporated into analyses. It is also important to ensure that participants have no alternative goals that could motivate responses toward the presumably devalued outcome (de Houwer et al., 2018). Moreover, researchers should consider the cognitive differences between goal-directed and habitual systems—particularly in terms of speed and cognitive demands—when designing tasks meant to measure their functioning. Habit tests that place no time or cognitive constraints on responses may easily allow participants to fall back on goal-directed control, even if stimulus–response habits were formed during training.

In conclusion, evaluating the validity of the measures we use as proxies for *habit strength* is essential. The idea that habits require extended training to develop and that they complement more flexible goal-directed control remains foundational to the construct and has been key in assessing the validity of habit measures (e.g., Hardwick et al., 2019; Luque et al., 2020; Martínez-Lopez et al., 2026). Recent claims that habits can be formed and expressed after limited training (Gera et al., 2023; Pool et al., 2022) should be taken with caution, as other, more parsimonious explanations are possible. Indeed, our findings suggest that “habit-like” responses observed in these paradigms likely reflect individual differences in the efficacy of outcome devaluation, rather than genuine habitual control.

## Supplementary information

Supplemental Material 1.

## Data Availability

All related data and materials of this research are openly available at the web repository: https://osf.io/tfhea/

## Footnotes

1 We first mirrored the analyses conducted by Pool et al. (2022), who had excluded the Obsessive-Compulsive Inventory (OCI-R) and the Beck Depression Inventory (BDI) scores due to having data from only a small subset of participants. However, in our subsequent analyses, we included these two measures.

2 Note that we used the pre–post differential response rate as the dependent variable; thus, the main effect of the cue was driven by the higher response rate for valued compared to devalued outcomes, specifically in the post-devaluation phase.

## Code availability

Analysis scripts and experimental programs used in the current study are publicly available at the web repository: https://osf.io/tfhea/
