# Supplementary material for: The evaluation of devaluation: Deficient outcome devaluation leads to wrongly considering goal-directed actions as habits
Source: Behav Res Methods. 2026 Jul 7;58(8):224. doi: 10.3758/s13428-026-03099-6 (PMC13342024; doi:10.3758/s13428-026-03099-6)
Supplement: Supplementary file 1 — Supplemental Material 1. [file 13428_2026_3099_MOESM1_ESM.pdf]

Supplemental Material:

The Evaluation of Devaluation: Deficient outcome devaluation leads to wrongly considering goal-directed actions as habits

Vázquez-Millán, A.[1,2], Martínez-López, P.[1,2], Rueda, M.[1], León, J. J.[1] and Luque, D.[1,2]

[1] Department of Basic Psychology, Faculty of Psychology and Speech Therapy, University of Malaga

[2] Instituto de Investigación Biomédica de Málaga y Plataforma en Nanomedicina-IBIMA, Plataforma BIONAND

A.2 Between-group comparisons in baseline questionnaire scores using the *Welch Two Sample t-test* (Minimal training vs. Overtraining).

Mean questionnaire scores for each training condition are displayed in Table 1, as well as the *t-test* results and their associated probability. We found no significant differences between training conditions in any of the ratings, so both groups of training were equivalent in the assessed psychological factors.

Table 1

Between-group comparisons of questionnaire subscales mean scores in each training condition

Questionnaire	Subscale	Mean score (SD)		*Welch Two Sample t-test*
		Minimal training (N = 54)	Overtraining (N = 51)	
Trier Inventory Chronic Stress (TICS)	Social overload (SOOV)	9.59 (4.77)	10.25 (4.75)	t (102.71) = -0.713, 95% CI [-2.51, 1.18]; p = 0.478
	Pressure to perform (PREPE)	16.52 (6.44)	16.22 (6.06)	t (103) = 0.248, 95% CI [-2.12, 2.72]; p = 0.804
	Work discontent (WODI)	12.98 (4.86)	11.59 (4.45)	t (102.9) = 1.534, 95% CI [-0.408, 3.19]; p = 0.128
	Excessive demands at work (EXWO)	10.22 (3.61)	9.55 (3.49)	t (102.94) = 0.972, 95% CI [-0.70, 2.05]; p = 0.333
	Lack of social recognition (LACK)	5.39 (2.94)	5.43 (2.71)	t (102.94) = -0.077, 95% CI [-1.13, 1.05]; p = 0.939

Questionnaire	Subscale	Mean score (SD) Minimal training (N = 54)	Mean score (SD) Overtraining (N = 51)	*Welch Two Sample t-test*
	Social Tensions (SOTE)	7.07 (4.38)	6.31 (3.77)	*t* (102.11) = 0.955, 95% CI [-0.82, 2.34]; *p* = 0.342
Trier Inventory Chronic Stress (TICS)	Social Isolation (SOIS)	9.85 (5.03)	9.51 (5.11)	*t* (102.43) = 0.345, 95% CI [-1.62, 2.31]; *p* = 0.730
	Chronic worrying (WORY)	9.02 (3.87)	8.78 (3.18)	*t* (101.05) = 0.340, 95% CI [-1.13, 1.60]; *p* = 0.735
	Work overload (WOOV)	18.09 (5.88)	16.8 (4.87)	*t* (101.29) = 1.225, 95% CI [-0.80, 3.37]; *p* = 0.223
Barrat Impulsivity Scale (BIS-11)	Attentional impulsiveness	21.54 (2.44)	21.10 (2.49)	*t* (102.36) = 0.911, 95% CI [-0.52, 1.39]; *p* = 0.364
	Motor impulsiveness	22.78 (4.31)	22.16 (4.65)	*t* (101.22) = 0.708, 95% CI [-1.12, 2.36]; *p* = 0.480

Questionnaire	Subscale	Mean score (SD)		*Welch Two Sample t-test*
		Minimal training (N = 54)	Overtraining (N = 51)	
	Non-planning impulsiveness	25.3 (4.59)	24.41 (4.59)	*t* (102.65) = 0.987, 95% CI [-0.89, 2.66]; *p* = 0.326
State-Trait Anxiety Inventory (STAI)	STAI-Trait	28.56 (9.16)	28.25 (9.94)	*t* (101.06) = 0.161, 95% CI [28.56, 4.01]; *p* = 0.873
Obsessive-Compulsive Inventory-Revised (OCI-R)	Global score	23.89 (11.33)	21.88 (12.85)	*t* (99.69) = 0.847, 95% CI [-2.69, 6.71]; *p* = 0.399
Beck Depression Inventory (BDI-II)	Global score	12.87 (8.54)	12.43 (7.94)	*t* (102.98) = 0.273, 95% CI [-2.75, 3.63]; *p* = 0.785

A.2 Internal consistency of each questionnaire's subscale, taking scores from the final whole sample.

We reported the value of the *Cronbach's alpha* and *McDonald's Omega* indexes about the internal consistency of each questionnaire dimension in Table 2.

Table 2

Internal consistency of subscales of the questionnaire in the sample used for this research

Questionnaire	Subscale	Cronbach's alpha (α)	McDonald's omega (Ω)
Trier Inventory Chronic Stress (TICS)	Social overload (SOOV)	0.86	0.87
	Pressure to perform (PREPE)	0.84	0.84
	Work discontent (WODI)	0.78	0.80
	Excessive demands at work (EXWO)	0.71	0.69
	Lack of social recognition (LACK)	0.77	0.73
	Social Tensions (SOTE)	0.82	0.80
	Social Isolation (SOIS)	0.85	0.84
	Chronic worrying (WORY)	0.85	0.82

Questionnaire	Subscale	Cronbach's alpha (α)	McDonald's omega (Ω)
	Work overload (WOOV)	0.86	0.86
Barrat Impulsivity Scale (BIS-11)	Attentional impulsiveness	0.45	0.52
	Motor impulsiveness	0.67	0.72
	Non-planning impulsiveness	0.64	0.69
	Global score	0.78	0.87
State-Trait Anxiety Inventory (STAI)	STAI-Trait	0.88	0.92
Obsessive-Compulsive Inventory-Revised (OCI-R)	Global score	0.87	0.92
Beck Depression Inventory (BDI-II)	Global score	0.92	0.96

B.1 Factor loading of each subscale inside the three main factors identified through *Exploratory Factor Analysis*.

Table 3 shows the factor loadings matrix to clarify how the subscales' scores are related to each of the three underlying latent factors: Impulsivity, Socio-occupational stress, and *Affective stress*. The factor solution was constructed based on these loadings.

Table 3

Factor loading matrix for the EFA solution of pre-registered analyses

Questionnaire	Subscale	Socio-occupational stress	Affective stress	Impulsivity
Trier Inventory Chronic Stress (TICS)	Social overload (SOOV)	**0.687**	-0.003	-0.137
	Pressure to perform (PREPE)	**0.769**	0.175	-0.257
	Work discontent (WODI)	**0.644**	0.115	0.324
	Excessive demands at work (EXWO)	**0.342**	0.369	0.115
	Lack of social recognition (LACK)	**0.794**	-0.108	0.062
	Social Tensions (SOTE)	**0.643**	-0.023	0.246
	Social Isolation (SOIS)	**0.460**	0.288	0.065

Questionnaire	Subscale	Socio-occupational stress	Affective stress	Impulsivity
	Chronic worrying (WORY)	0.171	**0.680**	-0.069
	Work overload (WOOV)	**0.444**	0.114	0.003
Barrat Impulsivity Scale (BIS-11)	Attentional impulsiveness	-0.070	0.375	**0.435**
	Motor impulsiveness	0.192	0.130	**0.559**
	Non-planning impulsiveness	-0.094	-0.214	**0.527**
State-Trait Anxiety Inventory (STAI)	STAI-Trait	-0.032	**0.961**	0.017

B.2 Fixed and interaction effects for each of the Linear Mixed Models run with the underlying latent factors obtained through EFA.

We report the beta coefficients, standard errors, confidence intervals, and *p-values* of all the fixed factors included in the model, as well as the interactions between them (Tables 4-6). These LMMs were made to evaluate the potential impact of the individual differences in the three main underlying factors extracted with EFA (Socio-occupational stress, Affective stress, Impulsivity) over the change in response rate provoked by the devaluation procedure.

Table 4

Fixed and interaction effects for LMM of the Socio-occupational stress factor

		Press Frequency			
	Predictors	Estimate β	SE	95% Confint	*p*
LMM Socio-occupational stress	(Intercept)	-0.100	0.060	[-0.218, 0.019]	0.101
	Group	-0.159	0.117	[-0.390, 0.071]	0.178
	Cue	-0.862	0.054	[-0.968, -0.756]	0.000
	Phase	-0.657	0.048	[-0.751, -0.564]	0.000
	SocStress	-0.004	0.060	[-0.121, 0.113]	0.947
	Trial order	-0.007	0.005	[-0.018, 0.003]	0.168
	Group * Cue	0.165	0.108	[-0.047, 0.376]	0.130
	Group * Phase	0.240	0.094	[0.056, 0.424]	0.012
	Cue * Phase	-1.752	0.107	[-1.962, -1.542]	0.000
	Group * SocStress	0.177	0.120	[-0.057, 0.412]	0.141
	Cue * SocStress	0.016	0.055	[-0.092, 0.123]	0.775
	Phase * SocStress	-0.054	0.048	[-0.147, 0.040]	0.264
	Group * Cue * Phase	0.251	0.215	[-0.170, 0.671]	0.246
	Group * Cue * SocStress	0.154	0.110	[-0.061, 0.370]	0.163
	Group * Phase * SocStress	0.141	0.096	[-0.047, 0.328]	0.144
	Cue * Phase * SocStress	0.011	0.109	[-0.203, 0.226]	0.916
	**Group * Cue * Phase * SocStress**	**0.179**	**0.218**	**[-0.249, 0.608]**	**0.413**

Note. SE = Standard Error; Confint = Coefficients with 95% Confidence Interval.; SocStress = Socio-occupational stress factor.

Table 5

Fixed and interaction effects for LMM of the Affective stress factor

		Press Frequency			
	Predictors	Estimate β	SE	95% Confint	*p*
LMM Affective stress	(Intercept)	-0.096	0.061	[-0.215, 0.022]	0.115
	Group	-0.160	0.118	[-0.391, 0.071]	0.177
	Cue	-0.859	0.054	[-0.965, -0.753]	0.000
	Phase	-0.655	0.047	[-0.747, -0.562]	0.000
	AfecStress	0.004	0.059	[-0.112, 0.120]	0.945
	Trial order	-0.007	0.005	[-0.018, 0.003]	0.168
	Group * Cue	0.166	0.108	[-0.046, 0.378]	0.127
	Group * Phase	0.238	0.093	[0.055, 0.420]	0.012
	Cue * Phase	-1.749	0.107	[-1.959, 1.539]	0.000
	Group * AfecStress	0.135	0.118	[-0.097, 0.367]	0.257
	Cue * AfecStress	0.007	0.054	[-0.099, 0.113]	0.896
	Phase * AfecStress	-0.076	0.047	[-0.167, 0.016]	0.110
	Group * Cue * Phase	0.254	0.214	[-0.166, 0.673]	0.239
	Group * Cue * AfecStress	0.144	0.108	[-0.069, 0.356]	0.187
	Group * Phase * AfecStress	0.107	0.094	[-0.076, 0.291]	0.255
	Cue * Phase * AfecStress	-0.033	0.108	[-0.244, 0.178]	0.758
	**Group * Cue * Phase * AfecStress**	**0.183**	**0.215**	**[-0.239, 0.604]**	**0.398**

Note. SE = Standard Error; Confint = Coefficients with 95% Confidence Interval.; AfecStress = Affective stress factor.

Table 6

Fixed and interaction effects for LMM of the Impulsivity factor

		Press Frequency			
	Predictors	Estimate β	SE	95% Confint	*p*
LMM Impulsivity	(Intercept)	-0.096	0.062	[-0.217, 0.025]	0.121
	Group	-0.175	0.120	[-0.409, 0.060]	0.147
	Cue	-0.862	0.055	[-0.969, -0.756]	0.000
	Phase	-0.657	0.048	[-0.751, -0.562]	0.000
	Impulsivity	0.048	0.061	[-0.072, 0.168]	0.438
	Trial order	-0.007	0.005	[-0.018, 0.003]	0.168
	Group * Cue	0.191	0.109	[-0.023, 0.405]	0.082
	Group * Phase	0.259	0.095	[0.073, 0.445]	0.007
	Cue * Phase	-1.759	0.108	[-1.970, -1.549]	0.000
	Group * Impulsivity	0.023	0.122	[-0.217, 0.263]	0.850
	Cue * Impulsivity	-0.080	0.056	[-0.190, 0.029]	0.154
	Phase * Impulsivity	-0.084	0.049	[-0.179, 0.011]	0.087
	Group * Cue * Phase	0.302	0.215	[-0.120, 0.724]	0.163
	Group * Cue * Impulsivity	0.063	0.112	[-0.155,0.282]	0.571
	Group * Phase * Impulsivity	0.044	0.097	[-0.147, 0.234]	0.654
	Cue * Phase * Impulsivity	-0.162	0.110	[-0.378, 0.054]	0.145
	**Group * Cue * Phase * Impulsivity**	**0.156**	**0.220**	**[-0.276, 0.587]**	**0.481**

Note. SE = Standard Error; Confint = Coefficients with 95% Confidence Interval.; Impulsivity = Impulsivity factor.

B.3 Reevaluating the moderating effects of individual differences when including the OCI-R and BDI questionnaires.

Consistent with the pre-registered analysis (Figure 1A), the EFA returned a three-factor model (Figure 1B). The distribution was the same for the previously included variables, mirroring the three above-mentioned factors: *Impulsivity*, *Socio-occupational stress*, and *Affective stress*. The two new measures of depression (BDI) and obsessive-compulsive symptoms (OCI-R) were assimilated into this last dimension of affective stress. In this case, the factorial solution was identical for the *oblimin* method and for the varimax rotation method. Applying the first one, the validity coefficients were $R^2_{Imp} = 0.83$, $R^2_{Sociocup} = 0.95$, and $R^2_{AffectStress} = 0.96$.

We performed a linear mixed model for each factor and found no significant interaction effects of Cue (Valued vs. Devalued) × Group (Minimal vs. Overtraining) × Devaluation (Pre vs. Post) × Factor Score (*Socio-occupational stress*: $\beta = 0.18$, SE = 0.22, 95% CI [-0.25, 0.61], p =.41; *Affective stress*: $\beta = 0.18$, SE = 0.22, 95% CI [-0.24, 0.61], p =.39; *Impulsivity*: $\beta = 0.17$, SE = 0.22, 95% CI [-0.26, 0.60], p =.44). Thus, we conclude that these individual differences are not influencing the change in response rates to valued and devalued cues before and after devaluation.

Figure 1

Factorial solutions for measures of individual differences

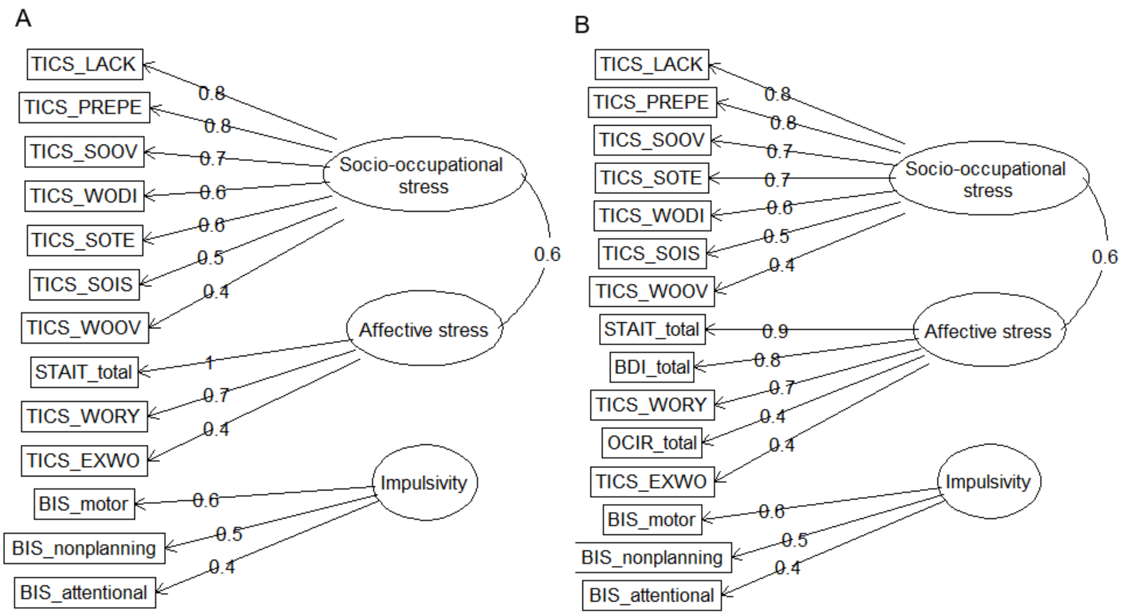

Note. **(A)** Results from pre-registered analyses using the same questionnaires as Pool et al. (2022). (**B**) Results from exploratory analyses when including the OCI-R and BDI scores.

B.4 Fixed and interaction effects for Linear Mixed Models conducted in exploratory analyses to evaluate the influence of devaluation efficacy on response rate changes.

We report the beta coefficients, standard errors, confidence intervals, and *p-values* of all the fixed factors included in the model, as well as the interactions between them (Tables 7-8). The LMM reported in Table 7 was made to evaluate the mediating effect of the outcome liking ratings over the change in response rate before and after devaluation for each cue. The second LMM (Table 8) introduced the cluster belonging and cue factors as predictors of the outcome liking ratings to check whether the devaluation procedure had differential efficacy depending on the cluster.

Table 7

Fixed and interaction effects of LMM evaluating the influence of outcome liking ratings over the press frequency change before and after the devaluation

	Press Frequency			
Predictors	Estimate β	SE	95% Confint	*p*
(Intercept)	0.156	0.086	[-0.012, 0.323]	0.071
Cue	-0.499	0.111	[-0.716, -0.283]	< 0.001
Phase	-0.409	0.094	[-0.593, -0.225]	< 0.001
OutcomeLiking	0.196	0.055	[0.088, 0.304]	< 0.001
Trial order	-0.007	0.008	[-0.022, 0.008]	0.345
Cue * Phase	-1.052	0.185	[-1.415, -0.689]	< 0.001
Cue * OutcomeLiking	0.540	0.133	[0.279, 0.801]	< 0.001
Phase * OutcomeLiking	0.384	0.087	[0.213, 0.556]	< 0.001
**Cue * Phase * OutcomeLiking**	**0.534**	**0.179**	**[0.184, 0.884]**	**0.003**

Note. The outcome liking ratings are considered a measure of the devaluation efficacy

Table 8

Fixed and interaction effects for LMM evaluating differences in outcome liking ratings between clusters of participants.

	Outcome Liking Ratings			
Predictors	Estimate β	SE	95% Confint	*p*
(Intercept)	0.028	0.024	[-0.019, 0.074]	0.249
Cue	-1.719	0.018	[-1.753, -1.684]	< 0.001
Cluster	0.169	0.048	[0.075, 0.262]	0.001
**Cue * Cluster**	**0.439**	**0.035**	**[0.370, 0.508]**	**< 0.001**

Note. The outcome liking ratings are considered a measure of the devaluation efficacy

C.1 Fixed and interaction effects of Linear Mixed Models conducted for re-analyses of Pool et al. (2022) and Gera et al. (2023) databases to evaluate the influence of devaluation efficacy on response rate changes.

We report the beta coefficients, standard errors, confidence intervals, and *p-values* of all the fixed factors included in the model, as well as the interactions between them (Tables 9-12). The LMMs reported in Table 9-10 were made to evaluate the mediating effect of the devaluation efficacy indexes, namely *Pleasantness Change Index* (PCI) and *Hunger Change Index* (HCI), over the change in response rate before and after devaluation for each cue, in the Pool and Gera datasets, respectively. This is, we introduced the press frequency as the dependent variable; whereas the Cue, Phase, and outcome liking and hunger scores were the predictors.

The next LMMs aimed to disentangle whether there were any differences between clusters of participants in how effective the devaluation was. Thus, we conducted one LMM per database for the hunger level variation, considering the Cluster belonging and the Phase factor as predictors (Tables 11-12); and another LMM (one for each database) to calculate whether the outcome liking ratings was influenced by the Cluster belonging, introducing the Phase and Cue factors as predictors as well (Tables 13-14).

Table 9

Fixed and interaction effects of LMM evaluating the influence of devaluation efficacy over the press frequency change before and after the devaluation in the Pool et al. (2022) database

	Press Frequency			
Predictors	Estimate β	SE	95% Confint	*p*
(Intercept)	0.073	0.133	[-0.189, 0.334]	0.586
Cue	-0.101	0.065	[-0.229, 0.026]	0.119
Phase	0.131	0.109	[-0.084, 0.345]	0.233
PCI	0.032	0.067	[-0.099, 0.162]	0.635
HCI	-0.072	0.079	[-0.227, 0.083]	0.365
Trial order	-0.023	0.004	[-0.03, -0.016]	<0.001
Cue * Phase	-0.235	0.062	[-0.356, -0.114]	<0.001
Cue * PCI	-0.085	0.033	[-0.149, -0.022]	0.009
Phase * PCI	-0.015	0.055	[-0.122, 0.092]	0.785
Cue * HCI	0.025	0.039	[-0.051, 0.1]	0.523
Phase * HCI	-0.249	0.065	[-0.377, -0.122]	<0.001
HCI * PCI	0.011	0.039	[-0.067, 0.088]	0.789
**Cue * Phase * PCI**	**-0.062**	**0.031**	**[-0.123, -0.001]**	**0.045**
**Cue * Phase * HCI**	**0.012**	**0.036**	**[-0.06, 0.083]**	**0.746**
Cue * HCI * PCI	-0.003	0.019	[-0.041, 0.034]	0.859
Phase * HCI * PCI	0.015	0.032	[-0.049, 0.078]	0.653
Cue * Phase * PCI *HCI	-0.042	0.018	[-0.078, -0.007]	0.019

Note. PCI = Pleasantness Change Index; HCI = Hunger Change Index.

Table 10

Fixed and interaction effects of LMM evaluating the influence of devaluation efficacy over the press frequency change before and after devaluation in the Gera et al. (2023) database

	Press Frequency			
Predictors	Estimate β	SE	95% Confint	*p*
(Intercept)	0.096	0.180	[-0.257, 0.45]	0.595
Cue	0.128	0.069	[-0.008, 0.264]	0.068
Phase	0.064	0.176	[-0.281, 0.409]	0.718
PCI	-0.042	0.103	[-0.244, 0.159]	0.683
HCI	-0.096	0.129	[-0.348, 0.156]	0.457
Trial order	-0.024	0.006	[-0.035, -0.013]	0.000
Cue * Phase	-0.246	0.086	[-0.414, -0.077]	0.004
Cue * PCI	0.185	0.040	[0.107, 0.262]	<0.001
Phase * PCI	0.127	0.101	[-0.071, 0.324]	0.210
Cue * HCI	-0.026	0.050	[-0.123, 0.071]	0.603
Phase * HCI	0.281	0.126	[0.034, 0.528]	0.028
HCI * PCI	0.045	0.069	[-0.091, 0.181]	0.520
**Cue * Phase * PCI**	**-0.333**	**0.049**	**[-0.43, -0.237]**	**<0.001**
**Cue * Phase * HCI**	**0.041**	**0.062**	**[-0.079, 0.162]**	**0.501**
Cue * HCI * PCI	-0.060	0.027	[-0.113, -0.008]	0.027
Phase * HCI * PCI	-0.093	0.068	[-0.227, 0.04]	0.173
Cue * Phase * PCI *HCI	0.090	0.033	[0.025, 0.155]	0.007

Note. PCI = Pleasantness Change Index; HCI = Hunger Change Index.

Table 11

Fixed and interaction effects of LMM evaluating differences between clusters in the pre-post variation of hunger levels (i.e., devaluation efficacy) in the Pool et al. (2022) database

	Hunger ratings			
Predictors	Estimate β	SE	95% Confint	*p*
(Intercept)	-0.238	0.034	[-0.304, -0.172]	<0.001
Phase	1.592	0.014	[1.565, 1.619]	<0.001
Cluster	0.032	0.067	[-0.099, 0.164]	0.629
**Phase * Cluster**	**-0.164**	**0.027**	**[-0.217, -0.11]**	**<0.001**

Table 12

Fixed and interaction effects of LMM evaluating differences between clusters in the pre-post variation of hunger levels (i.e., devaluation efficacy) in the Gera et al. (2023) database

	Hunger ratings			
Predictors	Estimate β	SE	95% Confint	*p*
(Intercept)	-0.191	0.064	[-0.317, -0.066]	0.003
Phase	1.173	0.021	[1.132, 1.213]	<0.001
Cluster	-0.090	0.128	[-0.341, 0.161]	0.485
**Phase * Cluster**	**0.264**	**0.041**	**[0.183, 0.344]**	**<0.001**

Table 13

Fixed and interaction effects of LMM evaluating differences between clusters in the specific pre-post variation of outcome liking scores for each cue (i.e., devaluation efficacy) in the Pool et al. (2022) database

	Outcome liking ratings			
Predictors	Estimate β	SE	95% Confint	*p*
(Intercept)	-0.196	0.027	[-0.249, -0.143]	<0.001
Phase	1.277	0.048	[1.182, 1.371]	<0.001
Cue	0.693	0.036	[0.622, 0.765]	<0.001
Cluster	-0.003	0.054	[-0.109, 0.103]	0.962
Phase * Cue	-1.511	0.020	[-1.551, -1.472]	<0.001
Phase * Cluster	0.071	0.096	[-0.118, 0.261]	0.460
Cue * Cluster	-0.140	0.073	[-0.283, 0.003]	0.056
**Phase * Cue * Cluster**	**0.587**	**0.041**	**[0.507, 0.666]**	**<0.001**

Table 14

Fixed and interaction effects of LMM evaluating differences between clusters in the specific pre-post variation of outcome liking scores for each cue (i.e., devaluation efficacy) in the Gera et al. (2023) database

	Outcome liking ratings			
Predictors	Estimate β	SE	95% Confint	*p*
(Intercept)	-0.203	0.048	[-0.298, -0.108]	<0.001
Phase	1.193	0.074	[1.047, 1.339]	<0.001
Cue	0.768	0.048	[0.674, 0.861]	<0.001
Cluster	0.091	0.097	[-0.099, 0.281]	0.348
Phase * Cue	-1.462	0.027	[-1.515, -1.409]	<0.001
Phase * Cluster	-0.267	0.149	[-0.559, 0.024]	0.075
Cue * Cluster	-0.428	0.095	[-0.614, -0.241]	<0.001
**Phase * Cue * Cluster**	**0.687**	**0.054**	**[0.581, 0.793]**	**<0.001**

C.2 Post-hoc comparison of the liking ratings by Cue (Valued vs. Devalued), Phase (Pre vs. Post Devaluation), and Cluster (Outcome-insensitive vs. Outcome-sensitive).

Results are disaggregated by the source database. We report the p-values without any adjustment method and with *Tukey's HSD* correction.

Changes in liking ratings before and after devaluation are displayed in Table 15. Both still-valued and devalued cues were significantly less appealing for participants after devaluation. This pattern is observed in *outcome-sensitive* and *outcome-insensitive* clusters, as well as in the two databases we explored. These results suggest that the devaluation procedure also affected the reward that was intended to preserve its value.

Before the devaluation occurred, there were no between-cluster differences in pleasantness ratings for valued and devalued outcomes in none of the cases (Table 2). Nevertheless, we observed significant differences in the post-devaluation phase. In the Pool et al. (2022) dataset, the *outcome-insensitive* cluster rated the still-valued cue as less appealing than its counterpart, the *outcome-sensitive* group (t = -2.630, SE = 0.097, p =.009, d = 0.294); although it loses the significance level when not applying *Tukey's HSD* correction. In the case of Gera et al. (2023), the difference is still more evident, as the *outcome-insensitive* cluster gave much higher liking scores to the devalued outcome, in comparison with the *outcome-sensitive* cluster (t = 3.769, SE = 0.162, p<.001, d = 0.666). This discrepancy emerged as significant even after applying the correction method. These results suggest that the efficacy of the devaluation procedure was quite different depending on each cluster of participants.

Finally, Table 3 shows that in the post-devaluation phase, the liking ratings were significantly higher for the valued cue compared to the devalued cue in both clusters;

whereas these differences were not present before the devaluation procedure. As an exception, looking at the Pool et al. (2022) analyses, the *outcome-sensitive* cluster initially scored as more pleasant the outcome that was going to be devalued in the posterior experimental phase ($t = -2.234$, SE = 0.026, $p = .026$, $d = 0.246$). It would mean this subgroup had some kind of baseline preference for that reward, although the significance did not survive the *Tukey's HSD* correction. Therefore, although the devaluation seemed to influence both outcomes (Table 1), these results suggest the effect was more pronounced over the targeted cue (i.e., *devalued outcome*).

Table 95

Pre vs. Post devaluation changes in outcome liking ratings by Cue × Cluster

OUTCOME LIKING RATINGS			Pool et al. (2022)		Gera et al. (2023)	
			VALUED	DEVALUED	VALUED	DEVALUED
PRE - POST	OUTCOME-SENSITIVE	*Estimated t-test*	4.089	25.858	3.608	18.974
		Standard Error	0.083	0.083	0.118	0.118
		Cohen's d	0.446	2.823	0.625	3.286
		Non-corrected p	<.001	<.001	<.001	<.001
		Corrected p (Tukey)	.001	<.001	.010	<.001
	OUTCOME-INSENSITIVE	*Estimated t-test*	13.042	35.620	5.142	16.643
		Standard Error	0.054	0.054	0.097	0.097
		Cohen's d	1.423	3.886	0.890	2.882
		Non-corrected p	<.001	<.001	<.001	<.001
		Corrected p (Tukey)	<.001	<.001	<.001	<.001

Table 10

Outcome-insensitive vs. Outcome-sensitive cluster differences in outcome liking ratings by Phase × Cue

OUTCOME LIKING RATINGS			Pool et al. (2022)		Gera et al. (2023)	
			VALUED	DEVALUED	VALUED	DEVALUED
OUTCOME-INSENSITIVE – OUTCOME-SENSITIVE	PRE DEVALUATION	*Estimated t-test*	1.632	-0.710	-0.811	-0.006
		Standard Error	0.067	0.061	0.104	0.098
		Cohen's d	0.184	0.080	0.144	0.001
		Non-corrected p	.104	.478	.419	.996
		Corrected p (Tukey)	.730	.997	.992	1
	POST DEVALUATION	*Estimated t-test*	-2.630	1.853	-1.037	3.769
		Standard Error	0.097	0.096	0.155	0.162
		Cohen's d	0.294	0.207	0.183	0.666
		Non-corrected p	.009	.065	.302	<.001
		Corrected p (Tukey)	.149	.584	.968	.006

Table 11

Valued vs. Devalued Cue differences in outcome liking ratings by Phase × Cluster

OUTCOME LIKING RATINGS			Pool et al. (2022)		Gera et al. (2023)	
			PRE	POST	PRE	POST
VALUED - DEVALUED	OUTCOME-SENSITIVE	*Estimated t-test*	-2.234	25.612	1.049	23.979
		Standard Error	0.026	0.065	0.07	.008
		Cohen's d	0.246	2.581	0.181	3.749
		Non-corrected p	.026	<.001	.367	<.001
		Corrected p (Tukey)	.335	<.001	0.966	<.001
	OUTCOME-INSENSITIVE	*Estimated t-test*	0.345	29.120	-0.087	17.117
		Standard Error	0.041	0.042	0.06	0.07
		Cohen's d	0.246	2.934	0.015	2.676
		Non-corrected p	.729	<.001	.988	<.001
		Corrected p (Tukey)	1	<.001	1	<.001
